# Bayesian model of signal rewiring reveals mechanisms of gene dysregulation in acquired drug resistance in breast cancer

**DOI:** 10.1371/journal.pone.0173331

**Published:** 2017-03-13

**Authors:** A. K. M. Azad, Alfons Lawen, Jonathan M. Keith

**Affiliations:** 1 School of Mathematical Sciences, Monash University, Clayton, VIC, Australia; 2 Department of Biochemistry and Molecular Biology, School of Biomedical Sciences, Monash University, Clayton, VIC, Australia; University of South Alabama Mitchell Cancer Institute, UNITED STATES

## Abstract

Small molecule inhibitors, such as lapatinib, are effective against breast cancer in clinical trials, but tumor cells ultimately acquire resistance to the drug. Maintaining sensitization to drug action is essential for durable growth inhibition. Recently, adaptive reprogramming of signaling circuitry has been identified as a major cause of acquired resistance. We developed a computational framework using a Bayesian statistical approach to model signal rewiring in acquired resistance. We used the *p*_1_-model to infer potential aberrant gene-pairs with differential posterior probabilities of appearing in resistant-vs-parental networks. Results were obtained using matched gene expression profiles under resistant and parental conditions. Using two lapatinib-treated ErbB2-positive breast cancer cell-lines: SKBR3 and BT474, our method identified similar dysregulated signaling pathways including EGFR-related pathways as well as other receptor-related pathways, many of which were reported previously as compensatory pathways of EGFR-inhibition via signaling cross-talk. A manual literature survey provided strong evidence that aberrant signaling activities in dysregulated pathways are closely related to acquired resistance in EGFR tyrosine kinase inhibitors. Our approach predicted literature-supported dysregulated pathways complementary to both node-centric (SPIA, DAVID, and GATHER) and edge-centric (ESEA and PAGI) methods. Moreover, by proposing a novel pattern of aberrant signaling called V-structures, we observed that genes were dysregulated in resistant-vs-sensitive conditions when they were involved in the switch of dependencies from *targeted* to *bypass* signaling events. A literature survey of some important V-structures suggested they play a role in breast cancer metastasis and/or acquired resistance to EGFR-TKIs, where the mRNA changes of *TGFBR*2, *LEF*1 and *TP*53 in resistant-vs-sensitive conditions were related to the dependency switch from *targeted* to *bypass* signaling links. Our results suggest many signaling pathway structures are compromised in acquired resistance, and V-structures of aberrant signaling within/among those pathways may provide further insights into the bypass mechanism of targeted inhibition.

## Introduction

Cell signaling pathways transduce input signals from extracellular to intracellular environments and determine various cell activities, including cell growth, proliferation, differentiation, migration, and apoptosis [1, 2]. Perturbation of a signaling network may occur when there are genetic alterations, such as DNA mutations and/or amplifications/deletions of a genomic region, or changes in gene expression (GE) [3, 4]. For example, the amplification or over-expression of the ErbB2 (HER2/neu) oncogene, that enhances various growth-related signaling activities [5] from receptor-level to effector-level [4], is commonly found in about 25% of breast cancer patients. In the majority of cancers, aberrant activities in signaling pathways are involved in various stages of tumor progression and metastasis [6–9].

Drugs targeting a signaling network, such as EGFR signaling pathway, often become ineffective as acquired resistance develops in cancer cells [10]. Primary reasons for acquired resistance to EGFR family receptor targeted therapies include: secondary mutations of targeted genes (e.g., the EGFR T790M mutation [11]), transcriptional and post-translational up-regulation of RTKs (Receptor Tyrosine Kinases) both within the receptor-family (e.g. ERBB3/HER3 [12, 13]) and other kinases (i.e. IGF1R, MET, FGFR2, FAK, SRC family kinases [14–16]), the over-expression of ABC transporters [3], and the re-activation of targeted pathways [5]. Moreover, tumor cells induce adaptive responses to targeted therapies [5] by *rewiring* in such a way that the adaptive signaling bypasses the inhibiting effects of initial treatments [4, 10, 17–19]. Therefore, rewiring of signaling networks plays a vital role as a non-genetic mechanism of acquired resistance [3, 14, 17, 18, 20]; targeting of which has the potential to improve the response durability of single kinase inhibitors [4, 5, 21]. However, reprogramming of signaling activities in acquired resistance inherently imposes increased uncertainties in the network structure when compared with their sensitive counterparts.

The functionality of biological networks is determined by their underlying architecture. Thus understanding, characterising, and analysing network structures are very important tasks in the field of systems biology [22]. Statistical modeling approaches offer a great deal of flexibility in terms of scalability and the number of local features that can be incorporated [22]. Moreover, as in other biological networks, signaling activities predicted using signaling data may be unreliable, whereas some crucial signaling links may not be predicted [23]. Measurements of the signaling activities often yield noisy data. Therefore, for such data-driven signaling networks a statistical modeling approach such as *exponential random graph models* (ERGMs) or p* can be a suitable choice [22, 23]. The *p*_1_-model, a special class of ERGMs which was originally proposed by Holland and Leinhardt [24], models the probability of an edge formation in the observed network based on network statistics (e.g. node degree) and associating model parameters with those statistics [22, 23].

Measuring the probabilistic nature of pair-wise relationships is an important aspect of modeling a gene-gene relationship network. Particularly in cancer drug resistance, some relationships between gene-pairs may evolve in the resistant conditions to compensate for the inhibiting effects of the drugs used [10, 19]. Some gene-pairs may have higher probabilities of evolving correlations in resistant conditions than in sensitive conditions. Simultaneously, some gene-pairs having high correlations in sensitive conditions may become loosely correlated (or even independent) in resistant conditions. For example, Komurov *et al.* reported that genes of the *glucose-deprivation response network* are up-regulated in lapatinib- (an EGFR/HER2 dual inhibitor) resistant conditions, thus providing an EGFR-independent mechanism of glucose uptake in cancer cells [19]. ErbB2-positive cancer cells largely depend on EGFR/ErbB2 signaling for their glucose uptake [19] which was recently reported as a major factor in oncogenic KRAS pathway mutations [25, 26]. Lapatinib mediates down-regulation of cell cycle machinery and up-regulation of cell cycle inhibitory complexes that are downstream of EGFR/ErbB2 signaling [19]. Moreover, the inhibitory effect of lapatinib on EGFR/ErbB2 signaling in the sensitive condition was found to be associated with glucose starvation of cancer cells, and thus induced cancer cell death [19]. However, in resistant conditions, up-regulation of activities involved in the *glucose deprivation response network* (and other hypoglycemic response networks) played an important role as a compensatory mechanism of glucose uptake in cancer cells for which tumors ultimately relapsed. Therefore, it can be hypothesized that genes involved in the process of cell proliferation and survival may evolve, in resistant conditions, to be highly correlated with the genes in the *glucose deprivation response network* in order to establish an alternate mechanism of glucose uptake in cancer cells, even though the inhibiting effects of lapatinib abrogated their dependencies on EGFR/ErbB2 signaling in sensitive conditions (See Fig 1 of [20].) Therefore, studying systematic characterizations of such differential dependencies among gene-pairs in resistant-vs-sensitive conditions, and their combined roles on particular genes’ dysregulations (in resistant-vs-sensitive) may reveal novel insights into mechanisms of acquired resistance.

Moreover, Komurov *et al.* [19] suggested that the drug resistance mechanism more likely occurs downstream of growth factor-mediated signaling pathways, such as Ras signaling, PI3K/AKT signaling, mTOR signaling, and others. However, an enormous number of diverse effector pathways may be involved in this process, making the prediction of biologically plausible hypotheses a challenging task. New computational approaches are needed to resolve such challenges in identifying the mechanistic underpinnings of acquired resistance.

Gene dysregulation is associated with aberrant signaling activities that are crucial for both cell growth and apoptosis in breast cancer [27]. For example, dysregulation [28] and/or mutation [28, 29] of apoptosis-related genes may overcome the initial response to apoptotic stimuli, thereby conferring resistance to apoptosis. Sharifnia et al. recently reported that several kinases and kinase-related genes from the Src family (e.g. *FGFR*1, *FGFR*2 and *MOS*) can compensate the loss of EGFR activity across multiple EGFR-dependent models [30]. Using unbiased gene-expression profiles of cells, their study revealed that over-expression of these EGFR-bypass genes plays a critical role in EGFR-independent activation of the MEK-ERK and PI3K-AKT signaling pathways in EGFR-mutant NSCLC cells. Recently, differential dependencies/associations were used to model rewiring in biological networks [31, 32]. Therefore, we hypothesize that differential associations between genes identified by modeling network reprogramming in resistant-vs-sensitive conditions could potentially explain gene dysregulation in acquired resistance.

**Fig 1:**
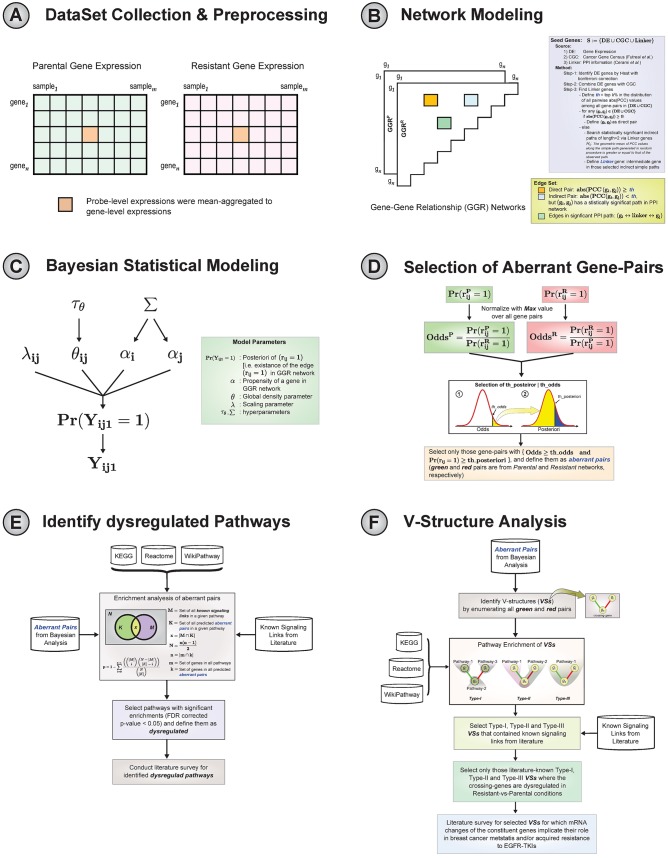
Schematic diagram of our proposed framework to identify and analyse aberrant signaling pathways in acquired resistance. (A) Gene expression datasets of breast cancer cell-lines for both parental and resistant conditions. (B) Two gene-gene relationship networks (GGR) were built from gene expression datasets of breast cancer cell-lines in Parental and resistant conditions. (C) & (D) A fully Bayesian approach was applied for detecting putative aberrant gene-pairs involved in acquired resistance. (E) Using the putative aberrant gene-pairs and a literature-curated signaling network, a statistical test was conducted to identify dysregulated pathways in acquired resistance. (F) Applying the known aberrant signaling links (from literature), we identify and explain the role of a proposed novel structure of aberrant pairs: V-structure (*VS*) in breast cancer metastasis and/or in developing acquired resistance to EGFR-TKIs.

In this study, we propose a computational framework to identify dysregulated signaling pathways in resistant-vs-sensitive conditions, and a possible mechanism of gene dysregulation in acquired resistance. The schematic diagram of our proposed framework is shown in Fig 1. We used two breast cancer cell-lines, SKBR3 and BT474, each having gene expression values measured under matched lapatinib-sensitive (parental) and lapatinib-resistant conditions. A gene-gene relationship network was constructed for each gene expression dataset by combining data-driven and protein-protein interaction (PPI) information indicative of both direct and indirect relationships between gene-pairs. Then we applied a fully Bayesian approach involving the *p*_1_-model to infer gene-pairs with differential posterior probabilities between these two conditions. Next, statistically significant dysregulated signaling pathways from KEGG, Reactome, and WikiPathway were identified by enriching putative aberrant pairs using literature curated signaling links. Finally, by proposing a novel pattern of aberrant pairs, called a V-structure, we identified possible mechanisms of dysregulation in resistant-vs-sensitive conditions that may be crucial for breast cancer metastasis and/or EGFR-TKI resistance. We hope such patterns revealed using our framework will lead to further insights into aberrant signaling activities in acquired resistance.

## Results

### A framework for identifying putative aberrant gene-pairs in acquired resistance

We developed a computational framework exploiting Bayesian statistical modeling to identify putative aberrant signaling links involved in acquired resistance. In this study, we hypothesized that aberrant signaling can be detected as differential probabilities of occurrence of gene-pairs in resistant-vs-parental conditions. Thus, after building gene-gene relationship networks individually from both parental and resistant conditions, a comparative study of edge probabilities in those two networks may reveal aberrant relationships due to acquired resistance.

Our framework constructs a gene-gene relationship network, *GGR*: = (*S*, *R*) by combining GE and PPI datasets, where *S* is a set of seed genes and *R* is a set of pair-wise gene relationships (Fig 1). Table 1 shows primary statistics for the *GGR* networks of both SKBR3 (GSE38376) and BT474 (GSE16179) cell-lines. For SKBR3 cell-lines (Parental and Resistant), we selected 897 seed genes comprised of 345 differentially expressed (DE) genes (Bonferroni corrected p-value ≤ 0.01), 370 genes from the Cancer Gene Census (CGC), and 502 and 479 linker genes from Resistant and Parental cell-lines, respectively. For BT474 cell-lines, we found 875 distinct seed genes comprised of 354 DE genes (Bonferroni corrected p-value ≤ 0.05), 357 CGC genes, and 477 and 489 linker genes from Resistant and Parental cell-lines, respectively. Note that to find DE genes in SKBR3 and BT474 cell-lines, two different p-value thresholds: 0.01 and 0.05 were used, respectively. This was done for two reasons: firstly, because the computational cost of using a conventional threshold of 0.05 with SKBR3 was prohibitive, and secondly, to ensure the numbers of DE genes in the two different cell-lines were comparable, and similarly for the sizes of the seed gene sets [for details see S1 Text].

**Table 1:** Primary statistics of Gene-Gene Relationship (*GGR*) network construction for both SKBR3 and BT474 cell-lines.

Cell Line	Cell Condition	# of DE Genes	# of CGC Genes	# of DE ∪ CGC Genes	# of All Pairs	# of Linker Genes	# of Total Seed Genes	# of combined Seed Genes	# of Direct Pairs	# of Indirect Pairs	# of PPI Pairs	# of Total Links
SKBR3	Resistant	345	370	704	247456	502	1262	897	49492	1440	1757	52560
	Parental					479	1245			1393	1758	52510
												
BT474	Resistant	354	357	698	243253	477	1100	875	48651	1572	1895	51998
	Parental					489	1101			1517	1951	51972

Our approach constructs a *GGR* in a series of stages: an initial set of genes is obtained by combining DE and CGC genes. Edges are added corresponding to direct relationships between pairs of these genes. We then search for indirect relationships among gene-pairs for which direct relationships couldn’t be found, and where indirect relationships are found the linker genes and the edges connecting them are added to the network. For the SKBR3 cell-line, the initial gene set contained 704 genes obtained by combining 345 DE and 370 CGC genes, whereas for the BT474 cell-line, the initial gene set contained 698 genes obtained by combining 354 DE and 357 CGC genes. To define direct relationships among the genes in the initial sets, we chose the top 20% from the ranked list of all pair-wise absolute Pearson Correlation Coefficients (PCC). Thus, we identified 49,492 (in both parental and resistant condition) and 48,651 (in both parental and resistant condition) direct relationships in SKBR3 and BT474 cell-lines, respectively. We justified this choice of threshold by applying an approach proposed by Elo *et al.* which analyses the topological properties of a co-expression network in order to find an optimal cutoff value [33] [for details see S1 Text]. In searching for indirect relationships, we found that 502 and 479 linker genes connect 1,440 and 1,393 distinct gene-pairs (for which direct relationships were not found) with the help of 1,757 and 1,758 distinct PPI links, for SKBR3 resistant and parental cell-lines, respectively. Similarly, for BT474 Resistant and Parental cell-lines, 477 and 489 linker genes connect 1,572 and 1,517 distinct indirect gene-pairs along with 1,895 and 1,951 distinct PPI links, respectively. In both datasets (SKBR3 and BT474), to build two *GGR* matrices for resistant and parental conditions with similar sets of genes, we constructed the final set of seed genes as an intersection of the two individual seed gene sets for Resistant and Parental conditions. Hence, 502 and 479 linker genes from SKBR3 resistant and parental conditions were combined with 704 (*DE* ∪ *CGC*) genes to form 1,262 and 1,245 seed genes, respectively, and then finding an intersection of these two sets yielded a set of 897 genes. Similarly, combining 698 (*DE* ∪ *CGC*) with 477 and 489 linker genes from BT474 resistant and parental genes produced 1100 and 1101 seed genes, respectively, and intersecting these resulted in a final set of 875 genes. At the end of this process, the SKBR3 resistant and parental *GGR* networks contained 897 distinct seed genes (*DE ∪ CGC ∪ Linker*) with 52,560 and 52,510 gene-gene relationships (*direct ∪ indirect ∪ PPI*), respectively, and the BT474 Resistant and Parental *GGR* network contained 875 distinct seed genes with 51,998 and 51,972 gene-gene relationships, respectively. Note that for both SKBR3 and BT474 cell-lines, although the total number of final seed genes is the same for both resistant and parental conditions, their respective *GGR* networks may contain different numbers of gene-gene relationships.

After building the *GGR* networks for both resistant and parental conditions ${Y_{k}}^{R}$ and ${Y_{k}}^{P}$ separately, we conducted Bayesian inference of parameters using the *p*_1_-model to estimate posterior probabilities of gene-gene relationships in each network. We used a WinBUGS script used in our previous work [10] for this inference. We ran the MCMC (Markov Chain Monte Carlo) method for 15,000 iterations, where the first 10,000 iterations were considered as ‘burn-in’, and the next 5,000 iterations were used for sampling. Time-series plots indicated that all parameters converged within the first few thousand iterations (data not shown). In both networks, the posterior probability of each edge was estimated to be the proportion of the 5,000 sampled networks in which that edge was present.

We identified a gene pair (*gene*_*i*_, *gene*_*j*_) as putatively aberrant if its posterior probabilities $Pr(Y_{ij1}^{R}=1)$ and $Pr(Y_{ij1}^{P}=1)$ of appearing in each network (resistant and parental networks, respectively) are significantly different. To determine which gene-pairs had this characteristic, we calculated two odds ratios—*Odds*^*R*^ and *Odds*^*P*^—as shown in Eqs (3) and (4) for each gene-pair (*gene*_*i*_, *gene*_*j*_). Note that since the two posterior probabilities used in these odds ratios may lie in different ranges, we normalized their values by dividing by their respective maximum values over all the gene-pairs in the respective sets. We then used two thresholds to define significance: first, we constructed the empirical distribution of odds ratios and chose only those gene-pairs which had odds ratios among the top 20%. For SKBR3 cell-lines, these threshold values were 2.53 and 1.66 for resistant and parental conditions, respectively, and for BT474 these values were 12.028 and 2.115, respectively. Next, we constructed empirical distributions of the posterior probabilities of the previously selected gene-pairs, and chose only those gene-pairs whose posterior probabilities were in the top 50% in their respective distributions. For SKBR3 resistant and parental cell-lines, these thresholds of posterior probabilities were 0.212 and 0.252, respectively, and for BT474 cell lines, 0.177 and 0.304, for resistant and parental conditions, respectively. More detailed explanations regarding these two types of thresholds are provided in the Supplementary Methods section in S1 Text. Thus, our framework finally selected 80,372 and 76,476 aberrant gene-pairs for SKBR3 and BT474 cell-lines, respectively, and we hypothesized that these aberrant gene-pairs have the potential to explain the mechanism of acquired resistance in breast cancer. Lists of all identified putative aberrant gene-pairs for both SKBR3 and BT474 cell-lines are shown in S1 Table.

#### Comparing posterior probabilities to correlation coefficients

**Fig 2:**
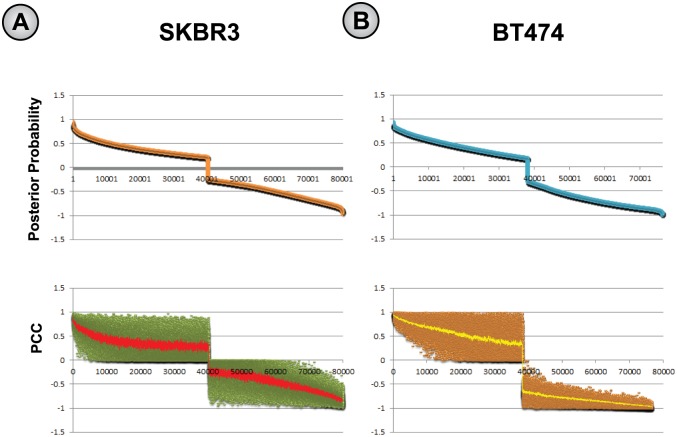
Comparing the posterior probabilities of putative aberrant gene-pairs with corresponding PCC (Pearson Correlation Coefficient) values that were defined among genes prior to the Bayesian analyses, (A) for SKBR3 and (B) BT474 cell-lines. The first figures in (A) and (B) show the sorted posterior probability values of the putative aberrant gene-pairs in descending order, and the second figures of (A) and (B) show the scatter plot of their corresponding PCC values. Note that for both the graphs in (A) and (B) the rank of ordered aberrant pairs is shown in X-axis, and the posterior probabilities and PCC values of *red* gene-pairs are shown in Positive Y-axis and those of *green* pairs are shown in Negative Y-axis, correspondingly. A trendline (red or yellow trendlines for the SKBR3 and BT474 cell-lines, respectively) is drawn for each of the scatter plots (in (A) and (B)) by using a moving average with a *window size* set to 25. For both SKBR3 and BT474, these trendlines clearly show the similarity of the signal contained in the PCC values (defined prior to Bayesian analyses) and the pattern of changes in *a posteriori* values (resulting from Bayesian analyses), and demonstrates the robustness of Bayesian statistical modeling for selecting putative aberrant gene-pairs involved in acquired resistance.

To investigate the robustness of our approach, we compared the posterior probabilities with the initial PCC (Pearson Correlation Coefficient) values for each of the putative aberrant gene pairs as shown in Fig 2. We treated the posterior probabilities of the *red* gene-pairs [see Methods] as positive values and the posterior probabilities of the *green* gene-pairs [see Methods] as negative, and plotted their sorted values in descending order (Fig 2). Next, we constructed a scatter plot with *corresponding* absolute PCC values for each of these gene-pairs, sorted based on posterior probabilities. We added a trendline using a moving average with *window size* 25, to investigate whether this trendline was in any way similar to the trend observed in the posterior probabilities. Interestingly, for both SKBR3 and BT474 cell-lines, the trendlines of PCC values revealed a visually similar pattern to that of the corresponding posterior probability values. This confirms our expectation that our Bayesian analysis is sensitive to a signal in the PCC values that would be otherwise difficult to detect.

### Many crucial signaling pathways are significantly enriched with aberrant gene-pairs in acquired resistance

**Fig 3:**
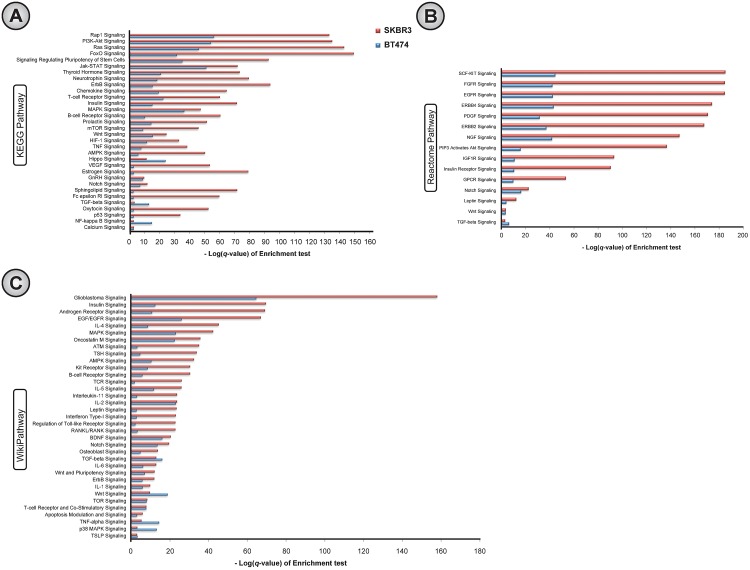
Analysis of dysregulated pathways by conducting pathway enrichment test of aberrant gene-pairs with known signaling links [34] involved in acquired resistance in SKBR3 and BT474 breast cancer cell-lines. Enrichments of all signaling pathways in (A) KEGG, (B) Reactome, and (C) WikiPathway.

To measure the significance of signaling pathways in terms of aberrant signaling activities in acquired resistance, we conducted a hypergeometric test. In this test, we measured how significant was the overlap between the set of literature-supported signaling links [34] found in a particular signaling pathway with the set of putative aberrant gene-pairs in the same pathway. We identified all the signaling pathways from KEGG, Reactome, and WikiPathway databases for which the corresponding *q*-value (FDR corrected *p*-value) from the above hypergeometric test was < 0.05 in both SKBR3 and BT474 cell-lines as is shown in Fig 3. For both SKBR3 and BT474 cell-lines, 71.11% (32 out of 45), 62.5% (15 out of 24), and 57.38% (35 out of 61) signaling pathways from KEGG, Reactome, and WikiPathways, respectively, were found to be significantly enriched with aberrant signaling gene-pairs in acquired resistance (Fig 3). Again, for all corresponding KEGG, Reactome, and WikiPathway databases, such high percentages of enriched signaling pathways found in both SKBR3 and BT474 cell-lines indicates that our framework is consistent in terms of finding aberrant gene-pairs in both cell-lines. Complete enrichment results of this hypergeometric test are reported in S2 Table.

We conducted a literature survey for the putative dysregulated signaling pathways, and found that the aberrant activities in most of these pathways are strongly associated with acquired resistance to EGFR tyrosine kinase inhibitors (EGFR-TKIs) [18]. EGFR (also known as HER1, or ErbB1) and EGFR 2 (also known as HER2/neu, or ErbB2) are cell surface transmembrane proteins, and members of the HER family of receptors. EGFR (in KEGG, Reactome, and WikiPathway) and ErbB2 (in Reactome) are reported to be frequently mutated and/or over-expressed in various types of cancer resulting in aberrant activities contributing to abnormal cell growth, survival, migration, and differentiation [35, 36]. However, over-expression and secondary mutations of both EGFR [11, 37–39] and ErbB2 [40] are associated with acquired resistance to EGFR-TKI. Moreover, being key components of cell signaling systems, these RTKs control major downstream signaling pathways, i.e. Ras/Raf/MAPK (in KEGG and WikiPathway), PI3K-Akt (in KEGG and Reactome), FoxO (in KEGG), and Jak-STAT (in KEGG) that are crucial for cancer cell growth and survival [3]. Moreover, as these downstream signaling pathways further regulate multiple downstream *effector* pathways (related to cell growth and survival), aberrant re-activation of those pathways provide a common mechanism to compensate for inhibition of targeted pathways, thereby conferring acquired resistance to EGFR-TKIs [4, 41, 42]. Interestingly, these signaling pathways (i.e. Ras, PI3K-Akt, FoxO, Jak-Stat signaling) were found as the top-most in the list of aberrant signaling pathways in both datasets (SKBR3 and BT474) based on the above hypergeometric test using KEGG database as shown in Fig 3. For ErbB4 signaling (in Reactome), recently it has been reported that in ErbB2-positive breast cancer cell-lines, ErbB4 was up-regulated at the protein level *in vitro* and re-activated PI3K-Akt signaling in resistant conditions compared to the sensitive condition, and the knock-down of ErbB4 induced apoptosis in both the lapatinib-resistant and trastuzumab-resistant cell-lines [43]. Rap1 (in KEGG) and ras (in KEGG) signaling are activated by lung cancer oncogene CRKL whose focal amplification (secondary mutation) was reported to be associated with acquired resistance to EGFR inhibitor [44]. Again, signals for cell proliferation and survival from activated AKT may transduce through several phosphorylated transcription factors, such as FoxO (in KEGG) [45], which indicates that the dysregulation of FoxO signaling pathway (in KEGG) may potentially be associated with resistance to EGFR-TKIs.

Our previous study found cross-talks between EGFR signaling and pathways triggered by other types of receptors, e.g. Notch, Wnt, IGF1R, GPCR, etc. which contributed to acquired resistance to lapatinib (an EGFR/Her2 dual inhibitor) [10]. Here, we also found these pathways showing significant aberrant activities in acquired resistance to lapatinib [Fig 3]. The activation of IGF1R signaling (in KEGG, Reactome, and WikiPathway) is commonly reported to induce acquired resistance to EGFR-TKIs by many studies [46, 47], and its inhibition could down-regulate PI3K-Akt signaling, eventually inhibiting cell growth, providing co-inhibition of EGFR and IGF1R signaling a clinical success [3]. Similarly, the Notch signaling pathway (in KEGG, Reactome, and WikiPathway) cross-talks with EGFR signaling in breast cancer, thus maintaining the cancer cell growth signal through MAPK and PI3K-Akt signaling [48]. It is suggested that an improved drug-sensitivity could be achieved by down-regulating the Notch signaling pathway with specific inhibitors [49, 50]. Again, genes involved in Wnt signaling (in KEGG, Reactome, and WikiPathway) were up-regulated in the resistant condition in both breast and colon cancer when compared to the sensitive condition [51, 52], thus contributing to acquired resistance to EGFR-TKIs [51].

Targeting angiogenesis is another important aspect of anticancer therapies [53], as aberrant vascularity and hypoxia are directly associated with tumor growth and survival [3]. In our analysis, we found aberrant angiogenic pathways including signaling by Vascular Endothelial Growth Factors (VEGFs) (in KEGG), Fibroblast Growth Factors (FGFs), and Platelet-Derived Growth Factors (PDGFs). It has been reported that the VEGF/VEGFR-2 feed-forward loop increases VEGF secretion in lung cancer via mTOR-dependent regulation that is required for the activation of downstream signaling [54], and the over-expression of VEGFR-1 reduces EGFR-TKIs sensitivity in different human cancer cells [3, 55]. Alternate activation of the FGFR signaling pathway (in Reactome) through the over-expressions of FGFR1 and FGF2 acts as a compensating mechanism for EGFR-TKIs [56] by maintaining signals for cell survival and proliferation in the downstream signaling pathways [4]. Again, it has been recently reported that, in PDGFR signaling (in Reactome), transcriptional de-repression of PDGFR-*β* contributed to compensating for the effects of EGFR-TKIs in EGFR-mutant glioblastoma via an mTORC1- and extracellular signal regulated kinase-dependent mechanism [21].

The hippo signaling pathway (in KEGG) is associated with cell proliferation, apoptosis, organ size control, and stem cell self renewal [57]. YAP is a transcription co-activator and oncoprotein [58], and plays a central role in cancer-related activities of the hippo signaling pathway [57]. Huang *et al.* have recently reported that down-regulating YAP expression in various cell-lines can improve the sensitivity of erlotinib (an EGFR-TKI) and cetuximab (anti-EGFR drug) [59]. We found the gene-pair AKT2:MYC as a signaling cross-talk between EGFR/ErbB and the TGF-*β* signaling pathway (in KEGG, Reactome, and WikiPathway) in our previous study [10]. Recently, it has been reported that combined inhibition of EGFR-TKIs (erlotinib) and TGF-*β* type I receptor inhibitor may improve sensitivity of EGFR-TKIs in lung cancer without EGFR T790M mutation [60].

For both SKBR3 and BT474 cell-lines, the primary findings in this study with supporting references are summarized in Tables 2 and 3. In this table, for each aberrant pathway, we also show what percentages of predicted gene-pairs from Bayesian analysis were previously defined as direct relationships, indirect relationships, and PPI during the network modeling. It is apparent that substantial proportions of predicted pairs came from direct and indirect relationships in both SKBR3 and BT474 cell-lines. This also indicates the robustness of our Bayesian modeling in inferring gene-pair relationships. Note that in the above calculation, if a predicted pair was defined both as direct and PPI, or both as indirect and PPI, then we counted that as direct or indirect, respectively, since that prediction for that particular pair was made by our framework. Again, some of the predicted pairs (by Bayesian modeling) may not be defined as direct or indirect previously, because the definitions of the terms *predicted* (based on *posterior probability* from Bayesian modeling), *direct*, and *indirect* were based on thresholds calculated from the distributions of corresponding values [see Methods]. Thus, the enrichment test with literature supported gene-dependencies [34] along with the evidences from the above literature survey confirm that our framework is able to identify significantly dysregulated signaling pathways that have key associations with acquired resistance in cancer.

**Table 2:** Summary of predicted dysregulated EGFR and its downstream signaling pathways from KEGG, Reactome and WikiPathway databases in acquired resistance in both SKBR3 and BT474 cell-lines.

Aberrant Pathways in EGFR-TKIs Resistance^k,r,w^	% of Direct Pair^(s,b)^k,r,w^^	% of Indirect Pair^(s,b)^k,r,w^^	% of PPI Pair^(s,b)^k,r,w^^	# of Enriched Pair^(s,b)^k,r,w^^	Enrichment q-value^(s,b)^k,r,w^^	Literature References
*EGFR and downstream pathways*						
EGFR signaling	(53.12%, 71.05%)^k^	(18.75%, 13.16%)^k^	—	(6, 32)^k^	(5.1e-16, 1.5e-94)^k^	[11, 37–39]
	(30.43%, 71.57%)^r^	(8.7%, 6.86%)^r^	(__, 0.49%)^r^	(18, 73)^r^	(3.2e-43, 1.7e-185)^r^	
	(56%, 71.79%)^w^	(12%, 7.69%)^w^	(__, 0.85%)^w^	(2, 4)^w^	(1.0e-26, 1.3e-67)^w^	
ErbB2 signaling	(33.96%, 74.12%)^r^	(7.55%, 4.12%)^r^	(__, 0.59%)^r^	(15, 64)^r^	(7.8e-38, 2.5e-168)^r^	[40]
ErbB4 signaling	(29.09%, 72.19%)^r^	(9.09%, 4.73%)^r^	(__, 0.59%)^r^	(17, 65)^r^	(6.6e-44, 7.5e-175)^r^	[43]
Ras signaling	(34.62%, 66.05%)^k^	(11.54%, 6.17%)^k^	(__, 0.62%)^k^	(22, 60)^k^	(6.5e-47, 6.9e-144)^k^	[4, 41–44]
MAPK signaling	(35.82%, 60.32%)^k^	(8.96%, 7.94%)^k^	—	(19, 23)^k^	(4.4e-37, 4.2e-48)^k^	[3, 4]
	(31.82%, 48.05%)^w^	(9.09%, 12.99%)^w^	—	(12, 19)^w^	(8.7e-24, 5.1e-43)^w^	
PI3K-Akt signaling	(35.27%, 61.85%)^k^	(10.62%, 5.69%)^k^	(__, 0.95%)^k^	(34, 75)^k^	(5.4e-55, 7.2e-136)^k^	[3, 4]
	(26.67%, 73.45%)^r^	(6.67%, 4.42%)^r^	(__, 0.88%)^r^	(6, 46)^r^	(1.5e-16, 1.4e-137)^r^	
Jak-Stat signaling	(25.49%, 71.19%)^k^	(19.61%, 13.56%)^k^	(__, 1.69%)^k^	(20, 7)^k^	(6.8e-52, 8.6e-73)^k^	[3, 4]
Rap1 signaling	(25%, 61.03%)^k^	(11%, 8.09%)^k^	(1%, 0.74%)^k^	(25, 53)^k^	(4.4e-57, 5.5e-134)^k^	[44]
FoxO signaling	(48.15%, 78.45%)^k^	(7.41%, 2.76%)^k^	(1.85%, 1.1%)^k^	(12, 54)^k^	(3.1e-32, 2.7e-150)^k^	[45]

^k^ KEGG

^r^ Reactome

^w^ WikiPathway

^s^ SKBR3

^b^ BT474;

**Table 3:** Summary of predicted dysregulated signaling pathways from KEGG, Reactome and WikiPathway databases that plays a role as compensatory pathway of EGFR/HER2 inhibition in acquired resistance in both SKBR3 and BT474 cell-lines.

Aberrant Pathways in EGFR-TKIs Resistance^k,r,w^	% of Direct Pair^(s,b)^k,r,w^^	% of Indirect Pair^(s,b)^k,r,w^^	% of PPI Pair^(s,b)^k,r,w^^	# of Enriched Pair^(s,b)^k,r,w^^	Enrichment q-value^(s,b)^k,r,w^^	Literature References
*Compensating Pathways of EGFR/HER2 inhibition*						
Notch signaling	(40%, 75%)^k^	(__, 25%)^k^	—	(2, 3)^k^	(8.6e-08, 1.7e-12)^k^	[48–50]
	(46.15%, 71.43%)^r^	(7.69%, 4.76%)^r^	—	(5, 7)^r^	(5.2e-17, 3.6e-23)^r^	
	(35%, 70.37%)^w^	(__, 7.41%)^w^	—	(5, 7)^w^	(3.2e-14, 3.2e-20)^w^	
Wnt signaling	(25%, 50%)^k^	(12.5%, 28.57%)^k^	—	(6, 8)^k^	(3.4e-16, 3.2e-25)^k^	[51, 52]
	(21.88%, 66.67%)^r^	(3.12%, 3.33%)^r^	—	(2, 2)^r^	(2.9e-04, 2.7e-04)^r^	
	(25%, 55.56%)^w^	(12.5%, 11.11%)^w^	—	(3, 6)^w^	(1.7e-19, 2.1e-10)^w^	
Insulin Receptor/IGF1R signaling	(40%, 70.49%)^k^	(13.33%, 9.84%)^k^	—	(6, 25)^k^	(7.9e-16, 3.1e-72)^k^	[3, 10, 46, 47]
	(29.41%, 87.93%)^r^	(5.88%, 3.45%)^r^	—	(4, 30)^r^	(1.3e-11, 6.6e-94)^r^	
	(35.9%, 80%)^w^	(12.82%, 9.33%)^w^	(__, 1.33%)^w^	(6, 28)^w^	(4.1e-13, 2.9e-70)^w^	
VEGFR signaling	(40%, 81.82%)^k^	(__, 4.55%)^k^	—	(1, 15)^k^	(1.9e-03, 3.6e-54)^k^	[3, 55]
FGFR signaling	(32.05%, 71.14%)^r^	(8.97%, 6.97%)^r^	(__, 0.5%)^r^	(18, 72)^r^	(7.6e-43, 1.7e-185)^r^	[4, 56]
PDGFR signaling	(40.78%, 71.35%)^r^	(3.88%, 3.78%)^r^	(__, 0.54%)^r^	(15, 67)^r^	(2.2e-32, 1.9e-171)^r^	[21]
*Others*						
Hippo signaling	(41.46%, 72.22%)^k^	(12.2%, 11.11%)^k^	—	(9, 4)^k^	(1.0e-24, 6.7e-12)^k^	[59]
TGF-*β* signaling	(22.22%, 100%)^k^	(11.11%, __)^k^	—	(4, 1)^k^	(1.1e-13, 5.0e-04)^k^	[10, 60]
	(50%, 50%)^r^	(25%, __)^r^	—	(2, 1)^r^	(4.4e-07, 7.7e-04)^r^	
	(54.55%, 30%)^w^	(18.18%, 40%)^w^	—	(5, 4)^w^	(1.1e-16, 1.2e-13)^w^	

^k^ KEGG

^r^ Reactome

^w^ WikiPathway

^s^ SKBR3

^b^ BT474;

#### Comparing with our previous study

**Fig 4:**
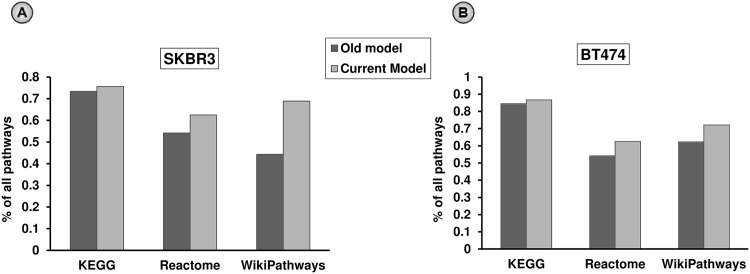
Performance comparison between the current model and our previous model [10] in terms of detecting perturbed signaling in acquired resistance. Percentages of signaling pathways detected as perturbed in acquired resistance by our *current* and *old models* in all KEGG, Reactome and WikiPathway databases: (A) in SKBR3, and (B) in BT474 cell-lines. For both the cell-lines, the performances using KEGG and Reactome pathways are comparable in both approaches, whereas our current model outperforms the old model for pathways from WikiPathway database.

To compare the performances of our current framework with our previous one [10], we investigated which of the two frameworks identify a greater number of dysregulated signaling pathways from KEGG, Reactome, and WikiPathway databases, since we used similar gene expression datasets (SKBR3 and BT474) in both approaches. We conducted a hypergeometric test to measure the statistical significance of the overlap between the aberrant pairs and known signaling links [34]. For that purpose, we defined the aberrant pairs in our previous approach [10] with *odds*^*P*^ and *odds*^*R*^ > 10.0, and posterior probabilities, $Pr(u_{ij}^{P}=1)$ and $Pr(u_{ij}^{R}=1)$ > 0.5. We found that greater percentages of pathways from KEGG, Reactome, and WikiPathway databases were found as perturbed (dysregulated) in acquired resistance when the current approach was used compared to the old one [Fig 4].

One of the main differences between these two approaches was in the definitions of the set of edges in *GGR* network models: the current approach used *direct* pairs and *non-direct* pairs (*indirect* pairs and *PPI* pairs), whereas the old approach only used *direct* pairs [10]. Therefore, we conducted two experiments to investigate the importance of non-direct pairs in the new model. First, in aberrant signaling pathways that were detected by our current but not the previous model, we observed what percentages of *enriched links* (i.e. aberrant pairs found as known signaling links) were previously defined as non-direct (indirect and PPI) pairs in our current model. In both SKBR3 and BT474 cell-lines, we found that all such dysregulated pathways from KEGG, Reactome, and WikiPathway databases contained high percentages of non-direct (indirect and PPI) *enriched links* [S3 Table]. Second, in aberrant signaling pathways that were detected by both of our current and previous models and were ranked (based on enrichment q-values) *high* in the current model but *low* in the previous model, we observed what percentages of *enriched links* were previously defined as non-direct in our current model. Considering the rank difference ≥ 10 (an empirical cutoff threshold), we found that aberrant pathways from KEGG, Reactome, and WikiPathway databases that showed such behavior in both SKBR3 and BT474 cell-lines, also contained high percentages of non-direct (indirect and PPI) *enriched links* [S4 Table]. Therefore, we claim that our current model demonstrate enhanced performances in detecting dysregulated signaling pathways in acquired resistance compared with our previous model.

#### Comparing with other methods

Next, we compared our framework with other published methods in terms of identifying the aberrant signaling pathways, specifically SPIA [61], DAVID [62], GATHER [63], ESEA [64] and PAGI [65]. The first three methods (i.e. SPIA, DAVID, and GATHER) are node-centric methods, where the role of differentially expressed (DE) genes was the key to identifying dysregulated pathways. However, ESEA and PAGI are edge-centric methods, where topological information regarding pathway structures was significantly exploited. All of these methods use GE datasets, except DAVID and GATHER which take a list of DE genes as input and identify aberrant pathways, or pathways enriched with given DE genes, respectively. For this comparative analysis, we used KEGG signaling pathways only, and for all the methods default configurations were applied unless specified otherwise.

**Fig 5:**
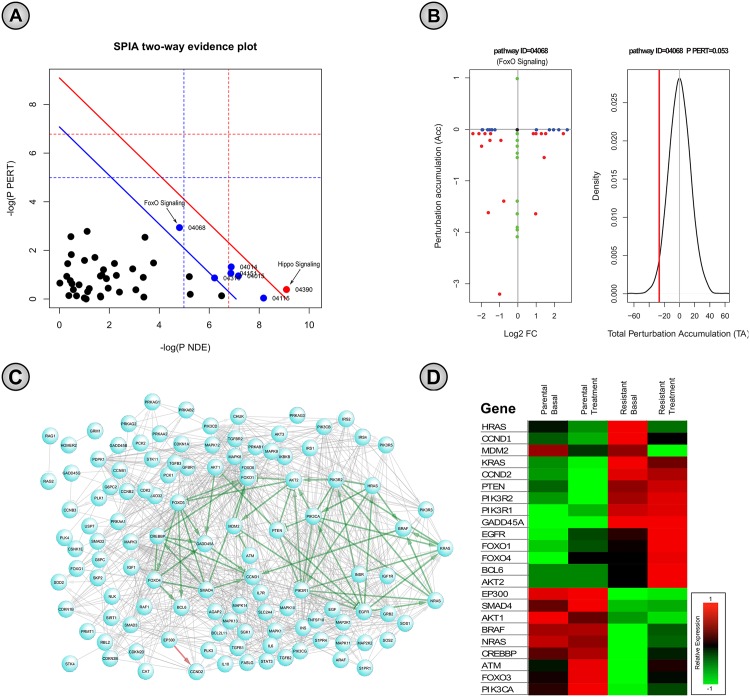
Detection of perturbed pathways with SPIA method. (A) Two-way evidence plot for all 45 KEGG pathways for BT474 cell-line is drawn. Here, pathways are represented with dots and the pathways with red dots and blue dots correspond to perturbed pathways with FDR-corrected and Bonferroni-corrected global *p*-value, *pG* < 0.05, respectively. (B) Next, the perturbation plot for FoxO signaling pathway (KEGG pathway ID = 04068) was also observed, since it contains the lowest perturbation *p*-value among all, *pPERT* = 0.053. In this plot, perturbation of all genes in the FoxO signaling pathway are shown as a function of their initial log2 fold-change (lower-left panel), where each dot indicates a gene in the pathway, and non-differentially expressed genes are assigned 0 as their log2 fold-change value. The null distribution and the observed net accumulated perturbation (red line) are shown in the lower-right panel. (C) Network view of FoxO signaling pathway for BT474 cell-line, where nodes are the constituent genes and the edges are known links collected from literature [34]. Here, *green* and *red* edges are the aberrant gene-pairs found by our method. (D) The heatmap of the genes’ expression in aberrant gene-pairs found by our method in FoxO signaling network for BT474 cell-line.

The SPIA method combines classical enrichment analysis and actual aberrant activities by analysing Cancer-Vs-Normal GE samples [61], and ranks corresponding signaling pathways by calculating a global pathway significance *p*-value, called *pG*. The global *p*-value (*pG*) is obtained by combining the perturbation probability (*p*-value: *pPERT*) and the probability of over-representation of DE genes (using log fold-change) in a particular pathway (*p*-value: *pNDE*) by using either Fisher’s method or the normal inversion method [61]. Here, we conducted the same analysis but with Resistant-vs-Parental GE samples aiming to capture the aberrant activities responsible for acquired resistance. In the case of multi-probe sets for the same gene, we used the most significant probe to get a single *log2* fold-change value per gene. For SKBR3 cell-lines, we found 5 signaling pathways as significant (raw *pG*-value ≤ 0.05) including Ras signaling, PI3K-Akt signaling, Rap1 signaling, hippo signaling, thyroid hormone signaling, and TGF-*β* signaling pathways. Interestingly, we found that the significance (−*log*(*q*-*values*)) of aberrant pathways found by our approach is strongly correlated with the global *p*-*values* (*pG*) found by SPIA analysis, both for all pathways (-0.4) and for above 5 signaling pathways only (-0.928). This indicates, in SKBR3 cell-lines, the signaling pathways from our framework with high enrichment of aberrant gene-pairs in acquired resistance are also consistent with the results from SPIA in terms of identifying aberrant activities. Again, for BT474, we found 12 signaling pathways with significant aberration (raw *pG*-value ≤ 0.05), i.e. hippo, p53, Ras, Rap1, PI3K-Akt, FoxO, Wnt, neurotrophin, insulin, estrogen, ErbB, and MAPK signaling pathways. Moreover, among these 12 signaling pathways, the first 6 had FDR-corrected *pG* ≤ 0.05, among which hippo signaling pathway had Bonferroni-corrected *pG* ≤ 0.05, as shown in Fig 5A. Among these 12 significantly dysregulated pathways in BT474 cell-line, we chose FoxO signaling to investigate further, since it was found highly perturbed by both SPIA (*pPERT* = 0.053) and our methods (enrichment *q*-*value* = 2.7 × 10^−150^). We observed perturbation plots for this signaling pathway (KEGG pathway ID = 04068), in which perturbations of all genes were plotted as a function of their initial *log*2 fold-change Fig 5B. Here, non-DE genes were assigned 0 for their *log*2 fold-change. However, many genes were identified as DE, since their absolute *log*2 fold-change values were mostly ∼2. Again, compared to the null distribution of net accumulated perturbation values, the observed value was also found significant as shown with the red vertical line in Fig 5B. Next, we also drew the network view of the FoxO signaling pathway, where the nodes were the constituent genes (from KEGG), and the edges were the known signaling links from the literature [34]. Here, we found 54 known signaling links that were also identified as aberrant gene-pairs by our method. Next, we plotted the heatmap of the expression values of the genes in these 54 known aberrant signaling links, where each expression value was the mean of all three replicates [66], z-transformed, and normalized with absolute max value (of the z-scores across the particular gene). Here, this heatmap not only shows the differential expression of the genes in aberrant gene-pairs but also indicates the similarities of their expression changes within this signaling pathway, which is a marker of aberrant activities in a modular way. Such differential gene expression in resistant-vs-parental conditions may indicate that pathway dysregulation within the signaling circuitry can be mediated by the corresponding aberrant gene-pairs.

As DAVID and GATHER both take as input a list of presumably differentially expressed genes for their pathway enrichments, we used the list of 703 and 683 distinct genes in the *list of aberrant gene-pairs* which were found by our framework from SKBR3 and BT474 cell lines, respectively. To detect statistically significant pathways using DAVID and GATHER we select those for which the raw *p*-*values* of their enrichment were < 0.05. For SKBR3 cell-line, DAVID and GATHER identified 15 and 5 signaling pathways as statistically significant, respectively. Again, for BT474 cell-lines they found 13 and 4 pathways as significant, respectively.

For both ESEA and PAGI analyses, we used our Resistant and Parental GE datasets for both SKBR3 and BT474 cell-lines. For both analyses, we used the default running parameters, except for the parameter *nperm* (the number of permutations) which was set to 1000. Both of these methods used a built-in set of topological structures of pathways from known pathway databases including KEGG. After running these methods with our GE datasets, if the identified signaling pathways had nominal *p*-*value* < 0.05, then we considered them as significantly dysregulated in resistant-vs-parental conditions. Thus, in the SKBR3 cell-line, we found 4 and 15 significantly dysregulated signaling pathways by ESEA and PAGI methods, respectively. For the BT474 cell-line, we found 2 and 12 signaling pathways significantly dysregulated by ESEA and PAGI, respectively.

All the dysregulated KEGG signaling pathways identified by any of these six methods are listed in Table 4. Some pathways were found consistently as dysregulated in both SKBR3 and BT474 cell-lines, but none were common to all six methods. However, our method identifies 33 KEGG pathways in both SKBR3 and BT474 cell-lines among which 17 were also identified by at least one of the other five methods (including both node-centric and edge-centric methods), for example MAPK, insulin (in DAVID, GATHER, and PAGI), ErbB, Wnt, B-cell receptor, Neurotrophin (in DAVID and PAGI), p53 (in DAVID and ESEA), and Jak-Stat signaling (in DAVID and GATHER). Moreover, our method identifies some novel dysregulated pathways in both SKBR3 and BT474 cell-lines which were not detected by any other methods. These pathways include Hif-1, AMPK, TNF and calcium signaling, which were reported to be involved in lapatinib-resistance in ErbB2-positive breast cancer cell-lines [4, 19, 67, 68]. Thus, the comparative identification of dysregulated pathways in resistant-vs-parental conditions in both SKBR3 and BT474 cell-lines indicates that our method is not only comparable to others but also able to detect novel findings which were validated by literature evidence.

**Table 4:** Comparative identification of pathway dysregulation in all 45 KEGG signaling pathways in resistant-vs-parental conditions in both SKBR3 and BT474 cell-lines. ‘S’ for SKBR3 cell-line, and ‘B’ for BT474 cell-line.

Pathway	SPIA	DAVID	GATHER	ESEA	PAGI	Our Method
MAPK signaling	B	SB	SB		SB	SB
Insulin signaling	B	SB	SB		SB	SB
ErbB signaling	B	SB			SB	SB
p53 signaling	B	SB		SB	B	SB
Wnt signaling	B	SB			SB	SB
Jak-Stat signaling		SB	SB			SB
B-cell receptor signaling		SB			SB	SB
Neorotrophin signaling	B	SB			SB	SB
Ras signaling	SB					SB
Rap1 signaling	SB					SB
Chemokine signaling		SB			S	SB
mTOR signaling		SB			B	SB
PI3K-Akt signaling	SB					SB
TGF-beta signaling	S	S			SB	SB
VEGF signaling		SB		S	S	SB
Hippo signaling	SB					SB
Fc epsilon RI signaling		SB				SB
Calcium signaling						SB
NF-kappa B signaling				S		SB
HIF-1 signaling						SB
FoxO signaling	B			S		SB
Phosphatidylinositol					SB	B
signaling system						
Sphingolipid signaling						SB
AMPK signaling						SB
Notch signaling		S			B	SB
Toll-like receptor signaling			SB		S	B
T-cell receptor signaling		B	S			SB
TNF signaling						SB
GnRH signaling					B	SB
Estrogen signaling	B					SB
Prolactin signaling						SB
Thyroid hormone signaling	S					SB
Oxytocin signaling						SB
Epithelial cell signaling in						B
Helicobacter pylori infection						
PPAR signaling					S	
cGMP-PKG signaling						B
cAMP signaling						B
Adrenergic signaling						B
in cardiomyocytes						
Hedgehog signaling			S			
signaling pathways regulating						SB
pluripotency of stem cells						
NOD-like receptor signaling					S	S
RIG-I-like receptor signaling					S	
Adipocytokine signaling				B		S
Glucagon signaling						B

### V-structures can explain the role of aberrant signaling in acquired resistance

#### The importance of V-structures

To investigate the potential of the putative aberrant gene-pairs to characterise acquired resistance, we hypothesized that *genes become dysregulated in acquired resistance because of the compensating effect of aberrant signaling that evolves in resistant-vs-parental conditions*. In the simplest cases, this will involve both *red* and *green* aberrant edges incident upon a particular dysregulated gene. To investigate this hypothesis, we identified all genes with at least two aberrant links to observe which of two possible architecture types are associated with a larger number of dysregulated genes: 1) both *red* and *green* aberrant edges incident upon a gene (forming V-structures—see Methods for the definition), or 2) only red or only green aberrant edges incident upon a gene. Next, we identified the dysregulated genes among these for which the following was true: a gene is over-/under-expressed (in any patient sample) in PT-vs-PB conditions, but respectively under-/over-expressed in both RB-vs-PB and RT-vs-PB conditions, where PB, PT, RB and RT stand for ‘Parental Basal’, ‘Parental Treatment’, ‘Resistant Basal’ and ‘Resistant Treatment’, respectively. The rationale for using only such combinations is as follows. Both expression datasets of SKBR3 (GSE38376) [19] and BT474 (GSE16179) [66] cell-lines contain *steady-state* measurements of signaling activities, for both parental and resistant conditions. Therefore, we hypothesized that the expression changes of dysregulated genes in PT-vs-PB conditions may indicate the sensitivity of Lapatinib drug (EGFR/HER2 dual inhibitor) in the parental (sensitive) conditions whereas the opposite changes in expressions in both RB-vs-PB and RT-vs-PB conditions may indicate two things: 1) the cell-line had already became resistant to the drug for which the tumorigenic phenotype of cancer cells relapsed in the resistant condition (RB-vs-PB), and 2) the resistance characteristics of the cell-line persisted even with further treatment with lapatinib (RT-vs-PB). For each comparison, we examined the *log*2 of fold-change values, and the treatment and basal doses were 1.0 *μ*M and 0 *μ*M, respectively. We found that, for both SKBR3 and BT474 cell-lines, higher percentages of dysregulated genes were identified with both *green* and *red* aberrant signaling links compared to those with only a single type of incident edge (either *red* or *green*). For SKBR3 and BT474 cell-lines we identified 111 and 108 genes with degree ≥ 2, respectively. For the SKBR3 cell-line, 90 of the 111 genes had only one type of aberrant signaling link incident upon them, out of which 48 showed dysregulation (53.3%), whereas the remaining 21 of the 111 genes had both *red* and *green* aberrant signaling links, out of which 13 genes were dysregulated (62%). Similarly, for BT474 cell-lines, among the 108 genes with degree ≥ 2, 78 out of 102 (76%) of genes with only one type of aberrant link and 6 out of 6 (100%) of genes with both types of aberrant signaling links, exhibited dysregulation. These results suggest that for a dysregulated gene in resistant-vs-parental conditions, the expression changes that occur upon treatment in parental conditions are likely to be compensated by aberrant signaling link(s) that evolved in resistant conditions. Therefore, the initial effect of inhibitors on oncogene(s)/tumor suppressor gene(s) becomes abrogated by restoring their tumorigenic phenotype once the cell acquires resistance to that inhibitor. This experiment demonstrates that V-structures can explain an interesting mechanism of acquired resistance in cell-lines by associating the dysregulated gene(s) with both *red* and *green* aberrant signaling links.

#### Type-II and Type-III V-structures provide a possible mechanism of gene dysregulation in acquired resistance

From the list of all putative aberrant gene-pairs (after Bayesian analysis), we enumerated all possible V-structures. We first listed all of the genes in *red* aberrant pairs, and separately listed all of the genes in *green* aberrant pairs. We then identified the genes common to both lists, which we termed *crossing*-*genes*. Next, we aggregated aberrant gene-pairs incident upon *crossing-genes* and enumerated all possible pairs of a *red* and *green* edge incident upon that gene. Thus, we found 23,156 distinct Type-I V-structures [see Methods for Type-I, Type-II and Type-III V-structure definitions] in SKBR3 cell-lines using signaling pathways from KEGG, Reactome, and WikiPathway, out of which 53 V-structures were found in the literature-curated signaling network [34]. Similarly for BT474, there were 5,271 distinct Type-I V-structures in all KEGG, Reactome, and WikiPathway signaling pathways, and 11 of them overlapped with the literature-curated network [34]. For Type-II V-structures in SKBR3 and BT474 cell-lines, 1,525 and 263 distinct V-structures were found in all KEGG, Reactome and WikiPathway databases, respectively, out of which 29 and 4 V-structures were found in the literature-curated network [34], respectively. For Type-III V-structures in SKBR3 and BT474 cell-lines, 940 and 376 distinct V-structures were found in all KEGG, Reactome, and WikiPathway databases, respectively, where 18 and 10 V-structures overlapped with the literature-curated signaling network [34]. A summary of these results for SKBR3 and BT474 cell-lines is provided in S5 and S6 Tables, respectively. Note that Type-I and Type-II V-structures have the potential to explain the role of signaling cross-talks in acquired resistance, but here we focus on Type-II V-structures only, since we have already investigated the role of signaling cross-talks in acquired resistance in our previous study [10] which are the similar kind of Type-I V-structures.

**Fig 6:**
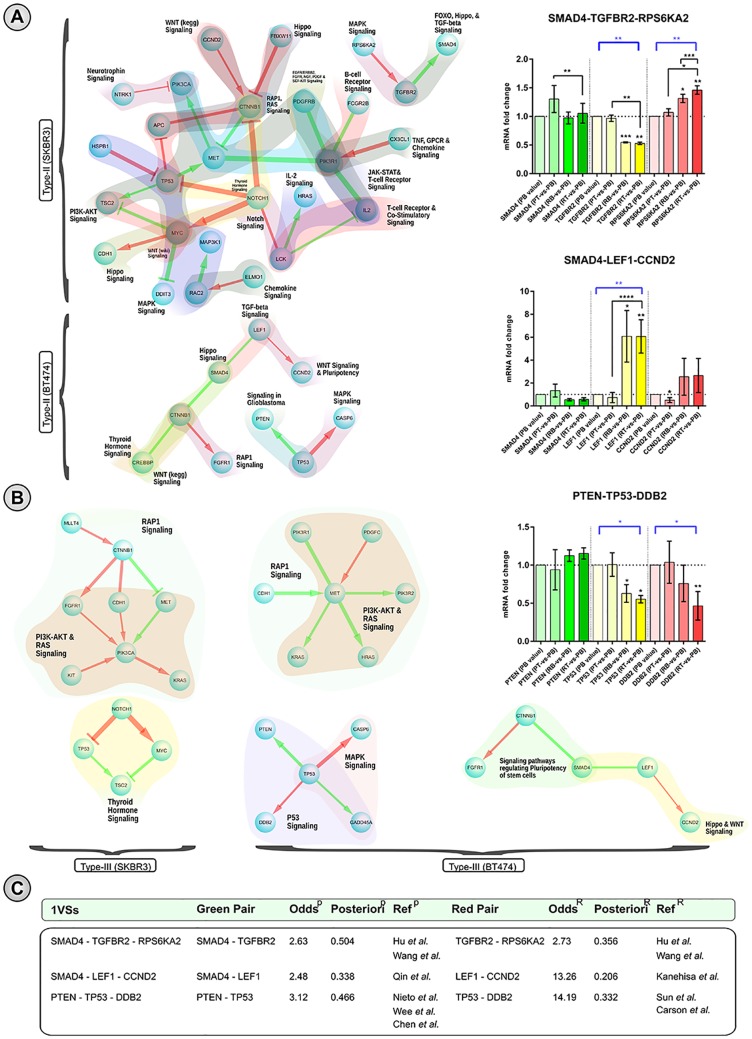
The role of literature-supported Type-II and Type-III V-structures (*VSs*) in explaining gene dysregulation in acquired resistance. (A) Network views of Type-II *VSs* along with their pathway annotations in SKBR3 and BT474 cell-lines. (B) Network views of Type-III *VSs* in SKBR3 and BT474 cell-lines. Note that *VSs* shown here are only those for which the crossing-genes were found as up- or down-regulated in PT-vs-PB conditions, but oppositely regulated in both RB-vs-PB and RT-vs-PB conditions. Nodes are genes, and the edges are known signaling links [34] that were also found as aberrant gene-pairs identified by our framework. Note that the width of edges is proportional to the posterior probability of corresponding pairs. Furthermore, for three *VSs* shown in (A) and (B) (right panels), mRNA changes for their constituent genes were found in the literature, implicating their role in breast cancer metastasis and/or in developing acquired resistance in EGFR-TKIs. (C) Above three *VSs* with their corresponding posterior probabilities, odds, and literature references of gene-pair associations for each of the *red* and *green* pairs. Statistical significance tests were done using t-tests and one-way ANOVA with multiple corrections (Sidak method). All the mRNA values were normalized by corresponding PB expression values in all three replicates. Significance was indicated by * (*p*-*value* < 0.05), ** (*p*-*value* < 0.005), and so on.

We investigated whether Type-II and Type-III V-structures can provide insights of a possible mechanism of acquired resistance in cancer cell-lines, focusing on the dysregulations of the *crossing-genes* in resistant-vs-parental conditions and its association with the GE changes of the other two genes in a particular V-structure. Our rationale was that the dysregulation of a *crossing-gene* may provide an indication that significant changes evolved in resistant-vs-parental conditions are associated with acquired resistance of cell-lines to a particular inhibitor. Moreover, significant GE changes in either of the two other genes (in the V-structure) would indicate that their differential associations with crossing-gene(s) may disrupt their functional coherence in signaling activities [30]. Therefore, we considered the above-mentioned 13 and 6 dysregulated genes in SKBR3 and BT474, respectively, for further analyses in which gene-pairs in corresponding V-structures overlapped with known signaling links [34]. Among the 13 dysregulated genes in SKBR3 cell-lines, 8 genes (*CTNNB*1, *TP*53, *MYC*, *RAC*2, *LCK*, *PIK*3*R*1, *PIK*3*CA*, and *TGFBR*2) were found in 22 (out of 29) literature-supported Type-II V-structures and 4 genes (*CTNNB*1, *TP*53, *MYC*, and *PIK*3*CA*) were found in 9 (out of 18) literature-supported Type-III V-structures (S5 Table). Similarly, among 6 dysregulated genes in BT474 cell-lines, 3 genes (*CTNNB*1, *LEF*1, and *TP*53) were found in 4 (out of 4) literature-supported Type-II V-structures and 4 genes (*MET*, *TP*53, *CTNNB*1, and *LEF*1) were found in 10 (out of 10) literature-supported Type-III V-structures (S6 Table). In Fig 6A, we show the network-view of the literature-supported Type-II V-structures incident upon the 8 and 4 dysregulated genes in SKBR3 and BT474 cell-lines, respectively, along with their annotated signaling pathways. Similarly, Fig 6B shows the Type-III literature-supported V-structures in both SKBR3 and BT474 cell-lines. Next, for each of the genes in the selected V-structures in Fig 6 we observed gene expression differences among all four conditions: PB (Parental Basal: 0 *μ*M), PT (Parental Treatment: 1.0 *μ*M), RB (Resistant Basal: 0 *μ*M), and RT (Resistant Treatment: 1 *μ*M) using both two-tailed paired t-tests and one-way ANOVA tests. For these statistical tests we used the mean expression value of all three replicates. In the t-tests, we compared the mean expression of all PT, RB and RT conditions with the mean of PB. Additionally, we also compared the mean of the RT condition with the means of the PT and RB conditions to observe 1) how a gene is behaving differently upon treatment in resistant-vs-parental conditions (RT-vs-PT), and 2) its expression changes upon treatment from its Resistant basal condition (RT-vs-RB). Moreover, one-way ANOVA tests (with the mean of PB as the control condition for the multiple comparison test) may indicate the significance of overall changes in all four groups. All of these statistical tests were done using GraphPad Prism 6.0 software. Concurrently, we also surveyed the literature to determine whether the observed significance of expression changes in resistant-vs-parental conditions were also supported by the literature. We found literature evidence (Fig 6C) supporting a role in breast cancer metastasis and/or in developing acquired resistance to EGFR-TKIs for the *SMAD*4 − *TGFBR*2 − *RPS*6*KA*2 (Type-II) V-structure in SKBR3, and *SMAD*4 − *LEF*1 − *CCND*2 (Type-II) and *PTEN* − *TP*53 − *DDB*2 (Type-III) V-structures in BT474 cell lines, respectively. Below we discuss these three V-structures in more detail.

- *SMAD*4 − *TGFBR*2 − *RPS*6*KA*2 (in SKBR3): *TGFBR*2 encodes a transmembrane protein which has been reported as a potent inhibitor of tumor growth and proliferation in breast epithelial cells, and loss of its function has also been associated with tumor malignancies [69]. Moreover, mRNA expression of *TGFBR*2 was reported to be significantly down-regulated in many tumorigenic cell-lines including SKBR3 and BT474 compared to the non-tumorigenic MCF-10F cell-lines [69]. This indicates the tumor-suppressing role of the *TGFBR*2 gene, and the reduction of its mRNA level may confer a resistance to targeted inhibitors by relapsing tumor growth and proliferation. In the GE dataset for the SKBR3 cell-line, the *TGFBR*2 gene was down-regulated in PT-vs-PB conditions without significance, but in resistant conditions it showed significant down-regulation compared to parental conditions (RB-vs-PB: *p*-*value* = 0.0003; RT-vs-PB: *p*-*value* = 0.002; RT-vs-PT: *p*-*value* = 0.001). A one-way ANOVA test also found the overall GE changes to be significant: Sidak corrected *p*-*value* = 0.0021. Thus, both literature evidence and GE data suggest an association of mRNA down-regulation of *TGFBR*2 gene with lapatinib resistance in SKBR3 cell-lines.
  *RPS*6*KA*2 (*RSK*3) encodes one of the members of the ribosomal S6 kinase which mediates resistance to PI3K pathway inhibitors in breast cancer [70]. RTK (Receptor Tyrosine Kinase) signaling induces the Ras and PI3K pathways, but upon lapatinib treatment such RTK signaling pathways are disrupted, downstream effectors (e.g. mTOR) are abrogated, and eventually Ras and PI3K signaling become inhibited [20]. Over-expression of *RSK*3 attenuates the apoptotic response and up-regulates protein translation, and thus promotes cell survival and proliferation under conditions of PI3K/mTOR blockade [70]. Moreover, lapatinib down-regulates the Akt pathway in both SKBR3 and BT474 cell-lines [71]. We observed significant and consistent over-expression of *RSK*3 mRNA in resistant condition compared to parental conditions in our SKBR3 cell-line dataset (RB-vs-PB: *p*-*value* = 0.011; RT-vs-PB: *p*-*value* = 0.0046; RT-vs-PT: *p*-*value* = 0.011; RT-vs-RB: *p*-*value* = 0.003). Overall expression changes were also found significant: Sidak corrected *p*-*value* = 0.0011. Therefore, both literature evidence and our experimental data strongly suggest that *RSK*3 over-expression is associated with lapatinib resistance via a PI3K/mTOR signaling blockade.
  *SMAD*4 is a downstream mediator of *TGF*-*β* [72] which plays an important role both in tumor suppression and progression in breast cancer [72, 73]. Liu *et al.* reported that *SMAD*4 expression was decreased in breast cancer cells compared to adjacent normal breast epithelial tissue [72]. Moreover, *SMAD*4 is sensitive to lapatinib according to the COSMIC database [74] with no mutational signature in breast cancer cell-lines. In our GE dataset of SKBR3 cell-lines, *SMAD*4 expression was up-regulated in PT-vs-PB, but was down-regulated in the RB-vs-PB condition, and again up-regulated in the RT-vs-PB condition. Note that however, that none of these comparisons were statistically significant in t-tests at the 0.05 level, and the one-way ANOVA also did not detect significant differences (Sidak corrected *p*-*value* = 0.101). Interestingly, both *SMAD*4 and *TGFBR*2 mRNA expression changes in PT-vs-PB conditions were non-significant; however, in resistant conditions (RB and RT) both *TGFBR*2 and *RPS*6*KA*2 showed significant changes in mRNA level compared to parental conditions (PB and PT). This may indicate the dependency switch of *TGFBR*2 from *SMAD*4 to *RPS*6*KA*2 in resistant-vs-parental conditions.
  *TGFBR*2 phosphorylates *SMAD*4 in the TGF-*β* signaling [34, 75], and both of their mRNA changes in parental conditions (PT-vs-PB) were non-significant. However, *TGFBR*2 is an upstream kinase that phosphorylates *RPS*6*KA*2 [34, 75], and both of their mRNA changes in resistant conditions were very significant compared to parental conditions. Thus, we hypothesize that the gene dysregulation of *TGFBR*2 in acquired resistance can be explained by its significant association with *RPS*6*KA*2 which evolved in resistant conditions compared to parental conditions.
- *SMAD*4 − *LEF*1 − *CCND*2 (in BT474): *LEF*1 plays an oncogenic role in breast cancer, since both mRNA and protein expression of this gene were found to be higher in breast cancer cell-lines compared to normal cells [76]. A high level of *LEF*1 was also found in *HER*2 expressing BT474 cell-lines [77], where *HER*2-activated *β*-*catenin* plays a crucial role in producing an increase in the downstream target *LEF*1 [76]. Increased expression of *LEF*1 drives cells towards resistance to TGF-*β*-induced growth inhibitory activities [78]. In our GE datasets of BT474 cell-lines, *LEF*1 mRNA expressions were significantly increased in resistant conditions compared to the parental basal condition (RB-vs-PB: *p*-*value* = 0.0178; RT-vs-PB: *p*-*value* = 0.003). Interestingly, over-expression of *LEF*1 was even more significant in resistant-vs-parental conditions in the presence of lapatinib (RT-vs-PT: *p*-*value* < 0.0001). Moreover, overall expression changes were also proved to be significant by one-way ANOVA test (Sidak corrected *p*-*value* = 0.004). Thus, the experimental data and the literature evidences support a role of *LEF*1 gene in lapatinib resistance in the BT474 cell-lines.
  *CCND*2 is involved in the cell cycle process, and is a regulatory subunit of a complex formed with *CDK*4 or *CDK*6 that is required for cell cycle G1/S transition [79]. Although *CCND*2 over-expression is found in ovarian, testicular [79] and gastric cancer [80], little is known about its role in breast cancer especially in the presence of lapatinib. In the GE data for the BT474 cell-line, *CCND*2 mRNA expression was significantly down-regulated in the PT-vs-PB condition (*p*-*value* = 0.024), and this possibly indicates the association of its mRNA down-regulation with lapatinib sensitivity in lapatinib-sensitive BT474 cell-lines. We investigated whether this behaviour is coherent with the literature. Schmidt *et al.* reported that both mRNA and protein expression of *CCND*1 and *CCND*2 were down-regulated when *FOXO*3*A* induced the process of cell cycle arrest [81]. Such inhibition of *CCND*1 and *CCND*2 perturbs *CDK*4 functionality to inactivate the S-phase repressor Rb [81]. Moreover, Hegde *et al.* reports that mRNA expression of *FOXO*3 and *CCND*1 were significantly up- and down-regulated, respectively, in both SKBR3 and BT474 cell-lines (lapatinib-sensitive) in response to lapatinib treatment [71]. To explain the above-mentioned down-regulation of *CCND*2, we observed *FOXO*3, *CCND*1 and *RB*1 mRNA changes in PT-vs-PB conditions (in BT474 datasets), to determine whether these are coherent with the above literature findings. In SKBR3 cell-lines, *FOXO*3 was significantly up-regulated (*p*-*value* = 0.0028) and *CCND*1 was significantly down-regulated (*p*-*value* = 0.0029). In BT474 cell-lines, 2 out of 3 replicates showed a similar pattern of mRNA changes for these two genes (*FOXO*3 and *CCND*1) (*p*-*values* = 0.042 and 0.017, respectively) as in SKBR3 cell-lines. In BT474 cell-lines *RB*1 mRNA expression was found slightly up-regulated in PT-vs-PB conditions. Moreover, *CCND*2 mRNA expressions are up-regulated in both resistant conditions (RB-vs-PB and RT-vs-PB) compared to the parental basal condition. The above experimental data may indicate the possible reason for *CCND*2 down-regulation in lapatinib-sensitive BT474 cell-lines with lapatinib treatment, and its mRNA up-regulation in both resistant conditions (RB-vs-PB and RT-vs-PB) could possibly be due to acquired resistance of BT474 cell-lines to lapatinib.
  *SMAD*4 expression was reported to be decreased in breast cancer cells [72], and the COSMIC database [74] reports *SMAD*4 as sensitive to lapatinib in the BT474 cell-line along with other EGFR-TKI, BIBW2992 and erlotinib [74] with *IC*_50_ *effect* = 0.225 (*p*-value = 0.000014) and with significant mutational signature in skin cancer, but none in breast cancer cell-lines. However, in the GE data for the BT474 cell-line, mRNA expression of *SMAD*4 was up-regulated in PT-vs-PB conditions, but was down-regulated in resistant-vs-parental conditions, with or without lapatinib treatment (RB-vs-PB and RT-vs-PT), indicating its sensitivity to lapatinib in parental conditions. Note that we observed no significant changes using a one-way ANOVA test (Sidak corrected *p*-*value* = 0.1212).
  SMAD4 binds to LEF1 [82], and the changes in expression of both of their mRNAs indicate sensitivity to lapatinib treatment in parental conditions (PT-vs-PB). Again, LEF1 regulates the transcription of *CCND*2 gene in the Wnt signaling pathway [83], and both genes exhibited up-regulation in resistant conditions compared to parental conditions. Thus, we can hypothesize that the dysregulation of the *LEF*1 gene can be explained by its differential associations with SMAD4 and CCND2 mRNA changes in resistant-vs-parental conditions.
- *PTEN* − *TP*53 − *DDB*2 (in BT474): *PTEN* is one of the most commonly mutated tumor suppressor genes, and the loss of its mRNA and protein expression are found in many metastatic malignancies including breast cancer [84]. *PTEN* modulates lapatinib sensitivity [85], and its loss acts as a marker of poor lapatinib response [58, 86, 87]. In the GE dataset for the BT474 cell-line, no mutation has been detected for *PTEN* and *TP*53 in their corresponding DNA sequences between parental and resistant conditions as reported in the original article associated with this dataset [66], and *PTEN* expression was up-regulated even in resistant-vs-parental conditions with or without lapatinib (RB-vs-PB, and RT-vs-PB), but the overall mRNA changes were not significant as tested with the one-way ANOVA test (*p*-*value* = 0.264). *TP*53 is another well known tumor suppressor gene, and its inhibition greatly inhibits apoptosis as p53 up-regulates several pro-apoptotic gene products including Puma, Noxa, Apaf-1, and Bax [88]. The loss of p53 is consistently associated with the acquired resistance of EGFR inhibitors cetuximab and erlotinib [89]. However, more experimental evidence is required to claim that p53 loss can be a predictive feature of acquired resistance to EGFR inhibitors [90]. In the GE dataset for the BT7474 cell-line, *TP*53 expression was significantly decreased in both RB-vs-PB (*p*-*value* = 0.013) and RT-vs-PB (*p*-*value* = 0.025) conditions, and the overall changes were statistically significant (Sidak corrected *p*-*value* = 0.01). For the *DDB*2 gene, its under-expression is correlated with poor outcome in ovarian cancer [91]. In breast cancer, although *DDB*2 showed putative oncogenic behaviour by promoting cell-cycle progression [92], it was not over-expressed in ER-negative breast cancer cells [92, 93], e.g. SKBR3 [93]. Moreover, *DDB*2 is down-regulated in lapatinib-resistant cell-lines [94]. This suppression was induced by the over-expression of the hepatitis B viral-encoded X protein (HBX) in the p53/lincRNA-p21 axis and IKK-dependent manner [94]. In our GE dataset for the BT474 cell-line, *DDB*2 was significantly down-regulated in resistant-vs-parental conditions (RT-vs-PB: *p*-*value* = 0.002) and the over-all changes were significant as well (Sidak corrected *p*-*value* = 0.046).
  p53 up-regulates or enhances *PTEN* transcription [95–97], and we found both genes’ mRNA changes in parental conditions (PT-vs-PB) to be non-significant. Moreover, p53 transcriptionally regulates *DDB*2 expression in a cell cycle-dependant manner [98, 99], and both of their mRNA changes were found to be significant, showing similar phenotypes in resistant-vs-parental conditions. Thus we can claim that the switch in dependency of *TP*53 from *PTEN* to *DDB*2 (in *PTEN* − *TP*53 − *DDB*2) can be a possible mechanism of *TP*53 dysregulation in acquired resistance.

Gene dysregulation plays an important role in developing acquired resistance to EGFR-TKIs in breast cancer [28–30, 100]. Here, along with literature-supported gene-gene associations in V-structures (Fig 6C), we demonstrated that the switch in dependency from the *targeted* signaling link involving *green* gene-pair (with the inhibitor) to the *bypass* signaling link involving *red* gene-pair (evolved in resistant conditions) is a possible mechanism mediating the dysregulation of *crossing-genes* in acquired resistance.

## Discussion

In this study, we proposed a computational framework that models signal rewiring by systematically characterizing potential aberrant signaling in acquired resistance. We hypothesized that an aberrant signaling link involved in acquired resistance may have differential probabilities of appearing (either higher, or lower) in resistant-vs-parental networks, where in each network, nodes were genes and the edges were the relationships among genes. In this gene-gene relationship network, called *GGR*, we considered both direct and indirect correlations (via *linker* genes) among genes for defining the edges that combine both data-driven (from gene expression) and topological (from PPI) information about gene-pairs. Note that the PPI edges in the statistically significant paths [see Methods], defining indirect relationships among gene-pairs for which direct relationships were not found, were also added to the final edge set [Table 1]. The rationale for including those PPI edges was: 1) to retain precise information regarding how indirect relationships were constructed, and 2) to better model the data-driven signaling networks (resistant and parental GGR networks) for the Bayesian statistical analysis (using *p*_1_-model) of their respective global structure formation. We used a fully Bayesian statistical model: a special class of Exponential Random Graph Model, called *p*_1_-model for inferring aberrant gene-pairs with differential posterior probabilities in resistant-vs-parental *GGR* networks, where these networks were constructed from matched gene expression values of resistant and parental conditions, respectively. When selecting aberrant gene-pairs, we chose the thresholds for *Odds* and posterior probabilities from their frequency distributions, sequentially. Firstly, we chose the gene-pairs with top 20% of odd-ratio values from two distribution individually (*odd*^*R*^ and *odd*^*P*^) by ensuring their mutual exclusivity after selection, and termed them as *red* and *green*, respectively. Then, we further filter *red* and *green* pairs with top 50% of their respective posterior probability values. Note that before calculating the *Odds* values, we normalized both posterior probabilities (from resistant and parental conditions) with their corresponding *max* values over all gene-pairs, individually, in order to achieve same scaling. All other model parameters in *p*_1_-model were estimated with Gibbs sampling [see Methods].

After detecting putative aberrant pairs in resistant-vs-parental conditions, we analyzed them in two-ways, 1) Identifying potentially dysregulated pathways in acquired resistance, 2) Identifying their roles in explaining a possible mechanism of acquired resistance via dysregulation of crucial genes. Using two lapatinib-treated breast cancer cell-lines: SKBR3 and BT474, our method was able to predict similar pathways as dysregulated. The rationale for using these datasets for our experiments was that—to the best of our knowledge—these are only datasets available for responsive and resistant lapatinib-treated ERBB2-positive breast cancer cell-lines. Our results suggested that signal rewiring is a major event in acquired resistance since we found a range of dysregulated pathways in both SKBR3 and BT474 cell-lines including EGFR-related pathways (e.g. EGFR, ErbB2, PI3K-Akt, Mapk, Jak-Stat, FoxO signaling, etc.) as well as other receptor-related pathways (e.g. Notch, Wnt, insulin, PDGFR, FGFR, VEGFR signaling, etc). Although there may be some false-positives in those results, we found literature evidence from Huang *et al.* [3] that aberrant signaling in most of our predicted dysregulated pathways were actually related with acquired resistance in EGFR-TKIs. Furthermore, our predictions of network re-adjustment in multiple signaling pathways were also consistent with the results recently published by Stuhlmiller *et al.* [5]. Their study suggested that multiple heterogenous kinases (e.g. DDR1, FGFRs, IGFI1, MET, etc.) compensate for the ErbB2 inhibition by kinome re-programming induced by lapatinib [5], which provides an indication that aberrant signaling activities in those kinase-related pathways are crucial for such bypass mechanism. Note that since the pathway annotations are still incomplete, we used three pathway databases here: KEGG, Reactome, and WikiPathways to define constituent genes of signaling pathways individually. However, to maintain the same true-relationship among those constituent genes we used literature-supported signaling links (collected from online resources of Wang Lab [34]) since it is the largest manually curated human signaling network as reported.

Gene dysregulation plays crucial roles in acquired resistance by mediating both uncontrolled cell-growth and disrupted apoptosis [27–29]. Here, to evaluate the potentialities of identified aberrant signaling, we conducted an analysis which demonstrated that the greater number of dysregulated genes were found in resistant-vs-parental conditions when they were incident with both *red* and *green*-types of aberrant pairs (V-structures) compared to those with single type only (either *red*, or *green*). Manual literature survey also validated some of the V-structures, such as *SMAD*4 − *TGFBR*2 − *RPS*6*KA*2, *SMAD*4 − *LEF*1 − *CCND*2, and *PTEN* − *TP*53 − *DDB*2, as consistent with our hypothesis. Thus, we claim that a mechanism of dependency shift from *targeted signaling* (by inhibitor) towards *bypass signaling* can potentially cause dysregulation of shared genes (crossing-genes). Similar idea of dependency switch was recently reported by Sharifnia et al. [30] that EGFR-dependent status of downstream signaling nodes can be modified by other over-expressed kinase-related genes that shared them (downstream signaling nodes) with EGFR-dependant signaling. However, to the best of our knowledge, our study is the first to emphasise the compensating effects of aberrant signaling upon mRNA expression changes of crucial genes by examining the dependency switch from *targeted* signaling to *bypass* signaling.

We included all the available genes from the Cancer Gene Census (CGC) into the list of seed genes in our framework for which gene expression data was available (see Methods): 370 and 357 genes in SKBR3 and BT474 cell-lines, respectively. Cancer genes are crucial for mediating various cancer related activities and many are hub genes in mammalian signaling networks [101]. Therefore, they are very important in terms of signaling network formation, an aspect which we examine in this study by statistical models (i.e. *p*_1_-model). Note that we combined cancer genes with a set of differentially expressed (DE) genes even though some may not be differentially expressed. However, cancer genes can still be important in network-based analyses of studies comparing two conditions (i.e. resistant-vs-parental). For example, in a network-based classification of breast cancer patients, Chuang *et al.* [102] reported that the subnetworks which can classify metastatic and non-metastatic patients contain genes playing a central role connecting DE genes even though those cancer genes were non-DE themselves [103]. Moreover, we intend to include all CGC genes, not just those ones that are breast cancer related, since no classifications are perfect, and the census is continuously being updated [104]. CGC genes are selected based on the mutational profiles of cancer patients [105], hence their transcriptional profiles may also reveal additional insights into the mechanisms of aberrant signaling activities in acquired resistance. To investigate the influence of CGC genes in our framework, we observed all the genes involved in all the V-Structures (VSs) of aberrant pairs (Type-I, Type-II and Type-III VSs) found in pathways from KEGG, Reactome and WikiPathway databases [See S5 Table]. We found that many of the genes involved in VSs overlapped with genes from CGC, where most of those cancer genes were not identified as DE genes during the formation of the seed gene list [see Methods] [S7 Table]. Thus, we claim that CGC genes were very important in the network-based analyses of our framework.

In this paper, we considered only gene expression values for modeling gene-gene relationship networks (*GGR*). However, we look forward to adapting other appropriate high-throughput datasets, such as miRNA expression, methylation, copy number aberration, and phosphorylation datasets into our framework in order to better model gene-gene dependencies in resistant-vs-parental conditions to reflect greater mechanistic insights. Moreover, the V-structures we have examined in our current study can be called *first-order* *V*-*Structures* since they involve only a single aberrant edge of each type (*red* and *green*). In future we intend to examine the role of higher order V-structures in acquired resistance.

## Materials and methods

### Literature and database search

Our research hypothesis was primarily focused on studying the acquired resistance mechanisms of HER2-positive breast cancer cells to lapatinib (an EGFR/HER2 dual inhibitor). Therefore, we conducted a literature survey in Pubmed database using keywords: ‘lapatinib’, ‘acquired resistance’, and ‘breast cancer’, which lead us to find two articles: Komurov *et al.* [19] and Liu *et al.* [66]. Both of these articles studied the resistance mechanisms of HER2-positive breast cancer cell-lines by analysing gene expression datasets of lapatinib-treated sensitive (parental) and resistant conditions. To find these gene expression datasets, we also searched GEO (Gene Expression Omnibus) database with the same keywords as above and found two data collections with accession IDs: GSE38376 and GSE16179, respectively. Detailed technical descriptions of cell-line preparation and dataset generation were reported in their respective original articles. The first dataset (GSE38376) included SKBR3 parental and resistant (SKBR3-R) cell-lines, and the second dataset (GSE16179) included BT474 parental and resistant (BT474-J4) cell-lines. In both of these datasets, expression values of both parental and resistant samples were measured first in basal condition (0 *μ*M), and then in treatment conditions (0.1 *μ*M and 1.0 *μ*M for GSE38376; 1.0 *μ*M only for GSE16179). For both GSE38376 (SKBR3) and GSE16179 (BT474), we converted probe-level expression values into gene-level values using the corresponding annotation files: GPL6947 (Illumina HumanHT-12 V3.0 expression beadchip) and GPL570 (Affymetrix Human Genome U133 Plus 2.0 Array), respectively, which were also collected from GEO database. For some genes, multiple probes were mapped to a single gene, and we averaged the GE values of such probes to obtain the final GE values of the corresponding gene. Next, for each collection (GSE38376 and GSE16179) we built two data matrices, one from the parental and another from the resistant GE dataset, where rows were labeled with gene symbols and columns were labeled with samples under different treatment conditions. A protein-protein interaction dataset was obtained from Cerami *et al.* [106]. For the enrichment analysis, we collected gene sets of all 1) the 24 signaling pathways from Reactome [107] (downloaded at 19/05/2014), 2) 45 signaling pathways from KEGG [83, 108] (downloaded at 12/05/2015), and 3) 61 signaling pathways from WikiPathway [109] (downloaded at 16/10/2014) databases. Each signaling pathway downloaded from these databases was encoded as tab-delimited lists of gene symbols. For KEGG signaling pathways, we built a parser program that extracted gene names from the web-responses after making HTTP web-requests to KEGG server using a list of IDs corresponding to signaling pathways.

### Constructing gene-gene relationship network

We denote the gene-gene relationship network as *GGR*:= (*S*, *R*) for each GE data matrix. Here, *S* is the set of seed genes, which is the union of a set of differentially expressed (DE) genes, a set of cancer genes collected from the Cancer Gene Census (CGC) [105], and a set of *linker genes* (see below) selected from the PPI network. *R* is the set of edges defined among the genes in the set *S*. The sets *S* and *R* were constructed as follows.

#### Defining *S*: The seed genes

We built the set *S* cumulatively; first a set of DE genes was identified by differential expression analysis of parental and resistant GE data using a two-tailed pooled Student’s t-test. For this test, significant *p*-values were identified using the Bonferroni correction method, and genes with such corrected *p*-values ≤ *threshold* (see Results) were selected as differentially expressed. Next, we added CGC genes for which corresponding GE data was available. The rationale for such inclusion is that CGC genes are well known to be hub genes in mammalian cellular signaling networks [101] where they play key regulatory roles in various cancer related activities. In the process of finding indirect relationships among (*DE* ∪ *CGC*) genes, a set of intermediate genes from the PPI network was identified, which we defined as *linker genes* (see next section). The final set of seed genes consisted of (*DE* ∪ *CGC* ∪ *Linker*) genes.

#### Defining *R*: The edges

To identify interacting gene pairs, all pair-wise absolute Pearson Correlation Coefficients (PCCs) were calculated for expression levels of the genes in the (*DE* ∪ *CGC*) gene set. The value demarcating the top 20% of absolute PCCs was selected as the threshold for defining *direct* relationships among the genes in the above set. That is, for each gene pair (*gene*_*i*_, *gene*_*j*_), if the corresponding PCC value was above the threshold then the pair was considered to have a *direct* relationship, and hence added into the set of edges, *R*.

Otherwise, a gene pair was said to have an *indirect* relationship if there was at least one statistically significant simple path in the PPI network between *gene*_*i*_ and *gene*_*j*_ via an intermediate gene (called a *linker* gene). Here, we imposed a path-length threshold of 2 and restricted to paths in the PPI network, otherwise considering all the remaining genes as possible intermediates would convert this searching procedure into an NP-hard problem. Simple paths of length 2 [for details see S1 Text] connecting a given pair (*gene*_*i*_, *gene*_*j*_) in the PPI network were considered statistically significant if one can reject the following null hypothesis: *the geometric mean of pairwise PCC values of constituent edges in the path is distributed as for paths of length 2 between these genes generated by a randomized procedure*. Random paths of the form *gene*_*i*_ → *linker* → *gene*_*j*_ were generated by replacing *linker* with any other gene in the network except *gene*_*i*_, *gene*_*j*_ and any gene on a path of length 2 connecting these genes in the PPI network. To evaluate the PCC for a random path, we used the same expression values for the genes as in the observed case. Paths were considered significant if the probability of generating a path using above randomized procedure with a geometric mean of pairwise PCC values greater than or equal to that observed for the PPI network was ≤0.05 (an empirical p-value). PPI edges comprising statistically significant simple paths were added to the set *R*. The set of edges *R* was finally composed of direct relationships, indirect relationships, and PPI edges of statistically significant simple paths, which are used for identifying those indirect relationships [see Discussion].

### Bayesian statistical modeling of *GGR* network

Exponential Random Graph Models (ERGMs) are parametric probability distributions over spaces of networks [24] that have been successfully used to evaluate probabilities of the presence of each edge in a network [23, 24]. Here, in order to infer edge probabilities in a gene-gene relationship network, we used the *p*_1_-model, a special class of ERGM introduced by Holland and Leinhardt [24]. The *p*_1_-model has previously been used by Bulashevska *et al.* [23] to model human protein-protein interaction networks. In this approach, edge probabilities are evaluated by summarizing topological properties of networks in a parametric form and associating them with sufficient statistics [23, 24]. The definition of the *p*_1_-model for a directed graph is contained in the original article [24]. An equivalent log-linear formulation was proposed by Fienberg and Wasserman [110], in which each directed edge was assigned four Bernoulli variables *Y*_*ij*00_, *Y*_*ij*01_, *Y*_*ij*10_ and *Y*_*ij*11_. Since our *GGR* network is an undirected graph, the model can be simplified by using only two Bernoulli variables *Y*_*ij*0_ and *Y*_*ij*1_ defined as follows:

$$\begin{array}{c} Y_{ijk}=\{\begin{array}{cc} 1 & if\;u_{ij}=k,\\ 0 & otherwise \end{array} \end{array}$$

where, the binary outcome *u*_*ij*_ = 1 if *gene*_*i*_ interacts with *gene*_*j*_ in *GGR*, and *u*_*ij*_ = 0 otherwise. Under this simplified model, the posterior probability of an edge connecting *gene*_*i*_ and *gene*_*j*_ is given by:

$$\begin{array}{c} log\{Pr(Y_{ij1}=1)\}=\lambda_{ij}+\theta +\alpha_{i}+\alpha_{j} \end{array}$$ (1)

$$\begin{array}{c} log\{Pr(Y_{ij0}=1)\}=\lambda_{ij} \end{array}$$ (2)

for *i* < *j*. Here, *θ* is the global density parameter, *α*_*i*_ is the expansiveness/attractiveness of *gene*_*i*_, and *λ*_*ij*_ is the scaling parameter ensuring $\sum_{k}^{}Y_{ijk}=1$. We hypothesized that some aberrant gene-pairs involved in acquired resistance may have unusually high probability of appearing in Resistant-vs-Parental conditions, whereas other pairs may have unusually low probabilities. Hence, we used two *Y*_*k*_ data matrices, ${Y_{k}}^{R}$ and ${Y_{k}}^{P}$, from *GGR* matrices of Resistant and Parental samples, respectively. Note that it is possible to replace the expansiveness and attractiveness parameters by a single parameter *α* that represents the propensity of a gene to be connected in an undirected network.

We used a fully Bayesian approach, both for modeling the network parameters and their estimation. To estimate the model parameters, we used Gibbs sampling, a Markov Chain Monte Carlo (MCMC) method implemented in WinBUGS [111] which allows users to construct complex Bayesian models in a simple manner. We constructed a hierarchical Bayesian model in which the model parameters were further defined as dependent upon hyperparameters as follows:

$$\begin{array}{cc} \theta ∼N(0,{\sigma_{\theta }}^{2}) & \\ \tau_{\theta }∼Gamma(a_{0},b_{0}) & \\ \left(\begin{array}{c} \alpha_{i}^{R}\\ \alpha_{i}^{P} \end{array})∼N((\begin{array}{c} 0\\ 0 \end{array}),\Sigma \right) & \\ \Sigma^{-1}∼Wishart((\begin{array}{cc} 1 & 0\\ 0 & 1 \end{array}),2) & \\ a_{0}=0.001 & \\ b_{0}=0.001 \end{array}$$

We assigned the density parameter *θ* a normal prior distribution with mean zero and standard deviation *σ*_*θ*_. (In fact, this was implemented in WinBUGS using the precision parameter $\tau_{\theta }={\sigma_{\theta }}^{-2}$). Next, the parameter *τ*_*θ*_ was assigned a gamma prior distribution with hyperparameters *a*_0_ = 0.001 and *b*_0_ = 0.001. We set *a*_0_ = 0.001 and *b*_0_ = 0.001 to express large uncertainty regarding the value $\sigma_{\theta }^{2}$, following [23]. For the propensity parameters $\alpha_{i}^{R}$ and $\alpha_{i}^{P}$, we selected the above prior following Adam *et al.* [112].

### Robust selection of aberrant gene-pairs

One of our primary hypotheses in this study is that aberrant gene-pairs involved in network re-wiring in drug-resistance are likely to have high probabilities of occurring in one network (resistant or parental) but low probabilities in the other network. To determine which gene-pairs exhibit this pattern, we calculated two odds ratios defined in the following equations:

$$\begin{array}{c} {odds}^{R}=\frac{Pr(Y_{ij1}^{R}=1)}{Pr(Y_{ij1}^{P}=1)} \end{array}$$ (3)

$$\begin{array}{c} {odds}^{P}=\frac{Pr(Y_{ij1}^{P}=1)}{Pr(Y_{ij1}^{R}=1)} \end{array}$$ (4)

where, $Y_{ij1}^{R}$ and $Y_{ij1}^{P}$ are defined for resistant and parental networks, respectively, and their corresponding posterior probabilities are estimated using MCMC sampling. Before calculating these ratios, we normalized the posterior probabilities by their respective maximum values over all gene-pairs, since two values ($Y_{ij1}^{R}$ and $Y_{ij1}^{P}$) may not be in the same scale. For the sake of brevity we refer to these ratios as odds ratios, but they are more appropriately called *normalized* odds ratios.

Our intention is to identify gene-pairs for which only one of the two odds ratios (Eqs (3) and (4)) is very high. Additionally, we require that both posterior probabilities exceed a minimum threshold, since very small denominators can yield high odds ratio scores even if the edge has low probability in both networks. We therefore defined two thresholds, one for odds ratio values and another for posterior probabilities. We examined the distributions of all *odds*^*R*^ and *odds*^*P*^ values and set a threshold demarcating the top 20%. Next, we examined the distribution of posterior probabilities for gene-pairs exceeding the odds ratio threshold and set a second threshold to demarcate the top 50%. Finally, we chose only those gene-pairs that had posterior probabilities above that threshold, and identified as putative aberrant gene-pairs that were potentially involved in network rewiring in acquired resistance.

Edges were then grouped into two types: gene-pairs for which the *odds*^*R*^ scores and the $Pr(u_{ij}^{R}=1)$ were greater than their respective thresholds in Eq (3) were defined as *red* pairs, whereas gene-pairs for which the *odds*^*P*^ scores and the $Pr(u_{ij}^{P}=1)$ were greater than their respective thresholds in Eq (4) were defined as *green* pairs.

### Enrichment of aberrant gene-pairs using known signaling links

Putative aberrant gene-pairs from the above Bayesian analyses were then further filtered by comparing them to another set of known (true) signaling links from the literature. For that purpose, we obtained a signaling network from the online resources of Wang Lab [34], which is claimed as the largest manually curated signaling network available to date. This network has more than 6,000 proteins and ∼63,000 binary relations defined, including activations, inhibitions and physical interactions. Note that signaling pathways from KEGG, Reactome, and WikiPathway databases were merely genesets, and to define true signaling links among the genes within those genesets we considered the signaling links from Wang Lab [34]. Next, to find dysregulated signaling pathways from KEGG, Reactome, and WikiPathway databases, we searched for significant overlaps between the set of true signaling links and the set of putative aberrant gene-pairs, for the genesets in a specific pathway. To this end, we designed a hypergeometric test as follows:

$$\begin{array}{c} p=1-\sum_{i=0}^{x-1}\frac{\left((\begin{array}{c} \left|M\right|\\ i \end{array})(\begin{array}{c} N-|M|\\ |K|-i \end{array})\right)}{\left(\begin{array}{c} N\\ \left|K\right| \end{array}\right)} \end{array}$$ (5)

where *N* is the number of distinct gene-pairs contained in all of the signaling pathways (from a particular pathway database) and all the predicted aberrant gene-pairs, *M* is the set of all known signaling links in a given pathway, *K* is the set of aberrant gene-pairs predicted by our framework, and *x* = |*M* ∩ *K*|. After measuring *p*-values using Eq (5), a False Discovery Rate (FDR) multiple comparison correction technique was conducted to obtain *q*-values. Signaling pathways with *q*-value <0.05 were considered to be significantly dysregulated in acquired drug resistance. A similar gene-pair enrichment test, called Edge Set Enrichment Analysis (ESEA) using the weighted Kolmogorov-Smirnov statistic was recently proposed by Han *et el.* for detecting dysregulated pathways in the context of gene expression datasets [113].

### Identifying V-structures

To investigate the role of signaling rewiring in acquired drug resistance, we searched for a configuration of edges that we call a *V-structure* (Fig 1F). A V-structure consists of three genes connected by one *red* edge and one *green* edge. One gene, called a *crossing-gene*, is connected to both of the other genes, to one by a *red* edge and to the other by a *green* edge. Thus V-structures involve both types of aberrant pairs: one gene-pair present only in Resistant conditions, and another gene-pair present only in Parental conditions, with the *crossing-gene* common to both pairs. Our rationale is that the compensatory kinases may switch the oncogenic-addiction of cancer-related (growth/survival) genes to overcome their dependencies upon their primary driver kinases (e.g. EGFR/HER2) that were initially targeted in parental conditions with inhibitors [19, 30], thereby relapsing into their tumorigenic roles in acquired resistance. We hypothesise that *crossing-genes* that are dysregulated restore their metastatic phenotype (i.e. up- or down-regulation of oncogenes or tumor suppressor genes, respectively) in resistant conditions by forming a V-structure in the rewired signaling network.

Therefore, we define a V-structure to be a pair of aberrant gene-pairs (*g*_*i*_, *g*_*k*_) and (*g*_*j*_, *g*_*k*_) such that (*g*_*i*_, *g*_*k*_) are connected by a *green* edge and (*g*_*j*_, *g*_*k*_) are connected by a *red* edge. To identify V-structures, first we identified the set of common genes in the two mutually exclusive sets of aberrant gene-pairs (*red* and *green* gene-pairs). This set of common genes are the *crossing-genes* (see Fig 1). Next, we observed and enumerated all the gene-pairs (*red* and *green*) incident on each of the *crossing-genes*, and enumerated all of the possible pairings of one *red* and one *green* edge to form a V-structure.

#### Pathway configurations of V-structures: Type-I, Type-II, and Type-III configurations

Next, for each V-structure, we identified signaling pathways from KEGG, Reactome, and WikiPathway databases that contained at least one gene in that V-structure. We classified V-structures into three sub-types based on their configurations relative to these pathways. Firstly, Type-I V-structures are those in which all three member genes belong to different signaling pathways. Type-II V-structures are those in which the two aberrant gene-pairs in a particular V-structure are from two different signaling pathways, with the *crossing-gene* common to both pathways. Type-III V-structures are those in which all three genes are from the same signaling pathway. Note that Type-I and Type-II V-structures may represent signaling pathway cross-talks that play crucial roles in acquired drug-resistance. In our previous study, we investigated and explained the concept of Type-I V-structures, their involvement in the cross-talk between EGFR/ErbB and other signaling pathways, and their contribution to lapatinib resistance [10]. Type-III V-structures can explain the aberrant co-regulation of genes *within a single pathway*. We observed and analysed all the V-structures that overlap with the literature curated signaling network [34].

## Supporting information

S1 Text Supplementary Text 1. Supplementary Methods.
(PDF)

S1 Table Supplementary Table 1. List of identified putative aberrant gene-pairs (for both SKBR3 and BT474) cell-lines in acquired resistance.
(XLSX)

S2 Table Supplementary Table 2. Full results of pathway enrichment tests of identified aberrant gene-pairs in acquired resistance from KEGG, Reactome, and WikiPathway databases for both SKBR3 and BT474 cell-lines.
(XLSX)

S3 Table Supplementary Table 3. Comparing our current model with the previous model by observing the percentages of non-direct (indirect and PPI pair) enriched links (aberrant pairs as known signaling links) in the aberrant signaling pathways from KEGG, Reactome, and WikiPathway databases *that were detected by our current but not the previous model*, for both SKBR3 and BT474 cell-lines.
(XLSX)

S4 Table Supplementary Table 4. Comparing our current model with the previous model by observing the percentages of non-direct (indirect and PPI pair) enriched links (aberrant pairs as known signaling links) in the aberrant signaling pathways from KEGG, Reactome, and WikiPathway databases *that were detected by both of our current and previous models, and were ranked (based on enrichment q-value) high in the current model but low in the previous model*, for both SKBR3 and BT474 cell-lines.
(XLSX)

S5 Table Supplementary Table 5. Summary of Type-I, Type-II and Type-III enrichment of V-structures in KEGG, Reactome, and WikiPathway databases in *SKBR3* cell-line.
(XLSX)

S6 Table Supplementary Table 6. Summary of Type-I, Type-II and Type-III enrichment of V-structures in KEGG, Reactome, and WikiPathway databases in *BT474* cell-line.
(XLSX)

S7 Table Supplementary Table 7. CGC genes in all the Type-I, Type-II and Type-III V-structures in *SKBR3* and *BT474* cell-lines.
(XLSX)
