# Supplementary material for: Bayesian model of signal rewiring reveals mechanisms of gene dysregulation in acquired drug resistance in breast cancer
Source: PLoS One. 2017 Mar 13;12(3):e0173331. doi: 10.1371/journal.pone.0173331 (PMC5348014; doi:10.1371/journal.pone.0173331)
Supplement: S1 Text — Supplementary Methods. (PDF) [file pone.0173331.s001.pdf]

Supplementary Text: Bayesian Model of Signal Rewiring Reveals Mechanisms of Gene Dysregulation in Acquired Drug Resistance in Breast Cancer

A. K. M. Azad[1,*], Alfons Lawen[2], Jonathan.Keith[1]
[1]School of Mathematical Science, Monash University
[2]Department of Biochemistry and Molecular Biology, School of Biomedical Sciences, Monash University
*E-mail: aaza7@student.monash.edu

Supplementary Methods

Parameter Selection

In this work, we used the following thresholds: *corrected* p-value thresholds for selecting differentially expressed genes, threshold for path length in the search of indirect relationships, cutoff thresholds for selecting direct relationships, odds ratio, and posteriori values. Detailed explanations and justifications behind these threshold selections are provided bellow. All other thresholds are p-value cutoffs for which a conventional value of 0.05 was used.

Selecting two different p-value thresholds for detecting differentially expressed genes in SKBR3 and BT474 cell-lines

For both SKBR3 and BT474 cell-lines, differentially expressed (DE) genes were selected based on Bonferroni-corrected p-values from a two-tailed pooled Students t-test. Genes that showed differential expression with corrected p-values $\leq$ threshold were selected as DE genes. By analysing gene expression datasets from parental and resistant conditions we identified 345 and 354 DE genes, for SKBR3 and BT474 cell-lines, respectively. The corresponding threshold values for corrected p-values were 0.01 and 0.05, respectively. It is true this implies SKBR3 DE gene selection was more stringent than that of BT474. This was done for two reasons: firstly, because the computational cost of using a conventional threshold of 0.05 with SKBR3 was prohibitive, and secondly, to ensure the numbers of DE genes in the two different cell-lines were comparable, and similarly for the sizes of the seed gene sets. Bayesian inference of model parameters (i.e. posterior probabilities of network edges) using MCMC sampling was the most time-consuming step in our whole framework. This step requires a longer period of time (considering the configurations of MCMC sampling) as the network size grows (i.e. as the number of seed genes increases). In this study, 0.01 and 0.05 p-value thresholds yielded 345 and 354 DE genes, and eventually 897 and 875 seed genes in total, for SKBR3 and BT474 cell-lines, respectively. We found that with these sizes of networks (both parental and resistant GGR networks) from SKBR3 and BT474 cell-lines, execution of the whole Bayesian inference procedure including MCMC sampling for 15,000 iterations and summarizing the monitored parameter (i.e. posterior probabilities of all node-pairs: of which there were 402,753 and 382,375 for SKBR3 and BT474 cell-lines, respectively) required more than one week, individually [data not shown]. Therefore, we feared that less stringent thresholds for SKBR3 (e.g. 0.05 yielding 135 additional DE genes) would result in an even larger GGR network for which the whole Bayesian inference step would run even longer. Therefore, we preferred to use the p-value threshold 0.01 for SKBR3 in order to produce a comparable number of DE genes, and thus a similar number of seed genes, so that we have the whole Bayesian inference procedure completed within a manageable time frame. Moreover, even after using a stringent threshold for SKBR3 cell-line our framework was able to identify high percentages of dysregulated pathways in acquired resistance: 75.56% (34 out of 45), 62.5% (15 out of 24) and 68.85% (42 out of 61) signalling pathways from KEGG, Reactome, and WikiPathways, respectively [S2 Table]. These results indicate that our framework demonstrated high performances in SKBR3 cell-line even with a more stringent p-value threshold. Therefore, we hypothesise that including a larger number of DE genes

by relaxing the threshold would yield little performance increase in terms of detecting aberrant signalling pathways in acquired resistance.

Selecting threshold for direct pair

Choosing a threshold for a co-expression network is a crucial step in network-based analysis since the representation of network structure, its functional relevance, and any network-based discoveries depend on the cutoff that is applied to all pair-wise co-expression values [1]. In this work, we applied a systematic approach proposed by Elo *et al.* [1] to identify an optimal cutoff threshold of such co-expression pairs (that is, *direct pairs*) by analysing the topological properties of a co-expression network. The approach is reported to achieve a balance between detecting as many biologically relevant co-expression links as possible, and controlling the *false-negative rates* [1]. It compares the clustering coefficient of the observed co-expression network and that of its randomized counterpart at a particular cutoff threshold point [1]. The clustering coefficient of a network is defined as follows:

$$C = \frac{1}{K} \sum_{k_i > 1} C_i \tag{1}$$

where $C_i = \frac{2E_i}{k_i(k_i - 1)}$ is the clustering coefficient of the node (gene) i, E_i denotes the number of edges between $k_i(> 1)$ first neighbours of node (gene) i [1]. Elo *et al.* hypothesised that the co-expression links omitted from the complete network by increasing the cutoff threshold value are more likely to be noise as long as the difference between the clustering coefficient of the observed network and its randomised counterpart is monotonically increasing [1]. Thus, a discrete optimization problem was formulated to find the optimal threshold C^* as follows:

$$C^* = \min_j \{r_j : C(r_j) - C_0(r_j) > C(r_{j+1}) - C_0(r_{j+1})\} \tag{2}$$

where the set of thresholds is: $r_0 < r_1 < ... < r_{J-1} < r_J$, $C(r)$ denotes the clustering coefficient of the co-expression network generated by applying the co-expression threshold r, and $C_0(r)$ is its randomised counterpart [1]. Here, $r_0 = 0$, $r_J = 1$, and $r_{j+1} = r_j + 1$ [1]. Elo *et al.* applied a configuration model in order to preserve the original degree distribution of the observed network [for details see Methods section of [1]]. Thus, the value of C_0 was formulated as follows:

$$C_0 = \frac{(\bar{k^2} - \bar{k})^2}{\bar{k}^3 N} \tag{3}$$

where $\bar{k} = \frac{1}{N}\sum_{i=1}^{N} k_i$, $\bar{k^2} = \frac{1}{N}\sum_{i=1}^{N} k_i^2$, and N = total number of nodes in the network [1]. This procedure define C^* to be the first *local maxima* in the $C - C_0$ curve [1]. In our study, by modelling *signalling rewiring* in resistant-vs-parental conditions, we identify system-level perturbations in the resistant condition compared to the parental condition. Consequently, we choose the threshold value for the parental condition by comparing to a random reference network and use the same number of pairs for the resistant condition in order to make a fair comparison. For each of the parental gene expression data sets in SKBR3 and BT474 cell-lines, we applied the above approach to identify an appropriate value of C^*. The resulting values of C^* were 0.62 and 0.74, which demarcate approximately the top 20% of pairs from the respective distributions of co-expression values in both SKBR3 and BT474 cell-lines. Therefore, we selected the top 20% of pairs to be considered as direct pairs in our analysis for all the GGRs. This reasoning has been added into the supplementary text.

Selecting thresholds for odds and posteriori

For selecting the thresholds of odds ratios (of posterior probabilities [see Methods in the original text]) of gene-pairs in all four cases: SKBR3-Parental, SKRB3-Resistant, BT474-Parental, and BT474-Resistant,

we made frequency distributions of all odds ratio values, respectively, from which we chose the top 20% gene-pairs, with odds ratio values of 1.66, 2.53, 2.16, 12.03 as cutoff thresholds ('*th_odds*'), respectively [Supplementary Figure 2]. Next, we constructed distributions of posteriori values of those selected gene-pairs, respectively, and chose the top 50% of gene-pairs from them with posteriori values of 0.252, 0.212, 0.304, and 0.177 as cutoff thresholds, respectively ('*th_posteriori*') [Supplementary Figure 3]. Although these two types of thresholds were chosen *empirically* from their respective distributions, applying them yielded two important outcomes. Firstly, for both SKBR3 and BT474 cell-lines, two sets of selected pairs from the top 20% of *parental* and *resistant* distributions [Supplementary Figure 2], respectively were *completely disjoint*, which is consistent with our methodological requirements that *red* and *green* [for definitions see original texts] aberrant pairs should be *mutually exclusive*. Secondly, mixtures of two distributions (assumed to be two clusters: left-cluster shown in *green* color and right-cluster shown in *violet* color) were clearly apparent in all individual scatter plots of *ODDs* VS *posterior probabilities* of gene pairs after applying '*th_odds*' in all four cases: SKBR3-Parental, SKRB3-Resistant, BT474-Parental, and BT474-Resistant [Supplementary Figure 4], and the top 50% pairs (from their frequency distribution) were residing in the right cluster (violet color) [Supplementary Figure 4] which is assumed to contain more important pairs than the left cluster (green color) because the posterior probability values of pairs in the former distribution are greater than in those of the latter.

Path length threshold in indirect relationships

In the search for indirect pairs for which no direct relationships were found, we applied an approach which exploits statistically significant PPI paths [see Methods in the original text] for which we constrained the length of those paths to be 2. We hypothesise that increasing this length threshold would impose additional computational costs and increase the time-complexity of the framework. We therefore chose the value of such path lengths to be 2, which is the minimum possible value involving single *linker genes* [see Methods in the original text].

References

1. Elo LL, Jarvenpaa H, Oresic M, Lahesmaa R, Aittokallio T. Systematic construction of gene coexpression networks with applications to human T helper cell differentiation process. Bioinformatics. 2007;23(16):2096–2103.

1 Supplementary Figures

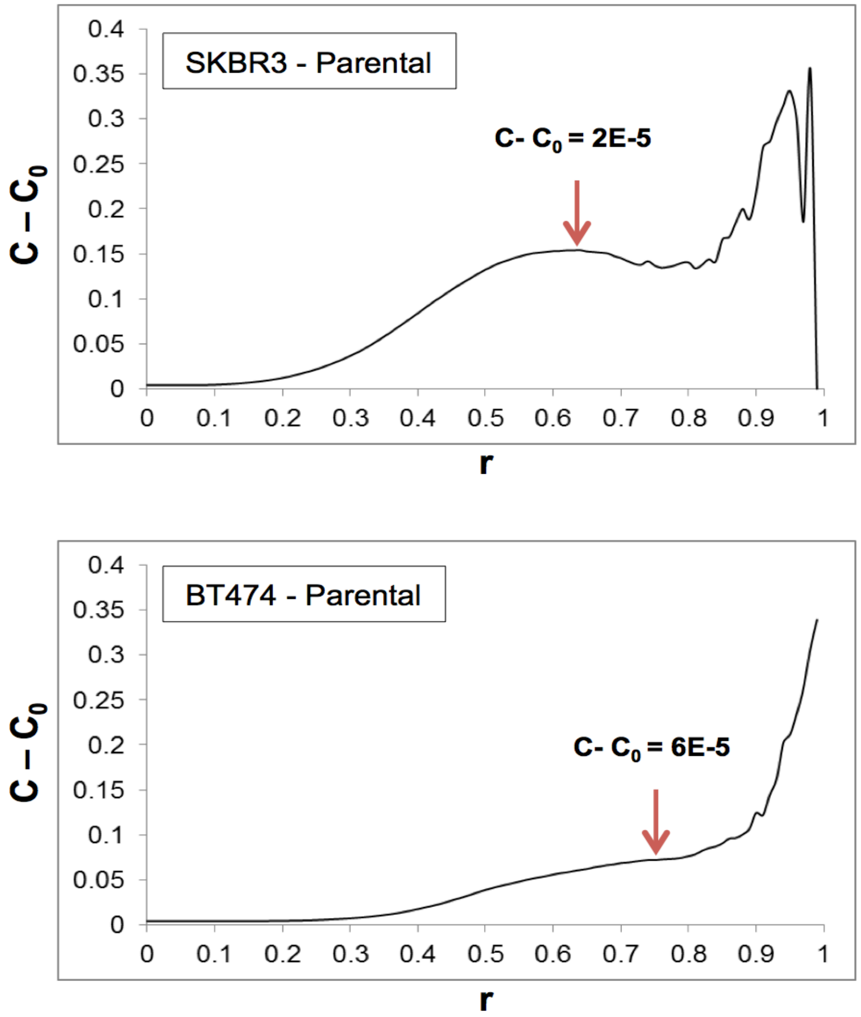

Figure 1. Behaviour of clustering coefficients (C) of observed co-expression networks against their randomized counterparts (C_0) at various co-expression value (absolute correlation), r. For each gene expression dataset, first *local maxima* in the $C - C_0$ is shown at the continuous case with red arrow.

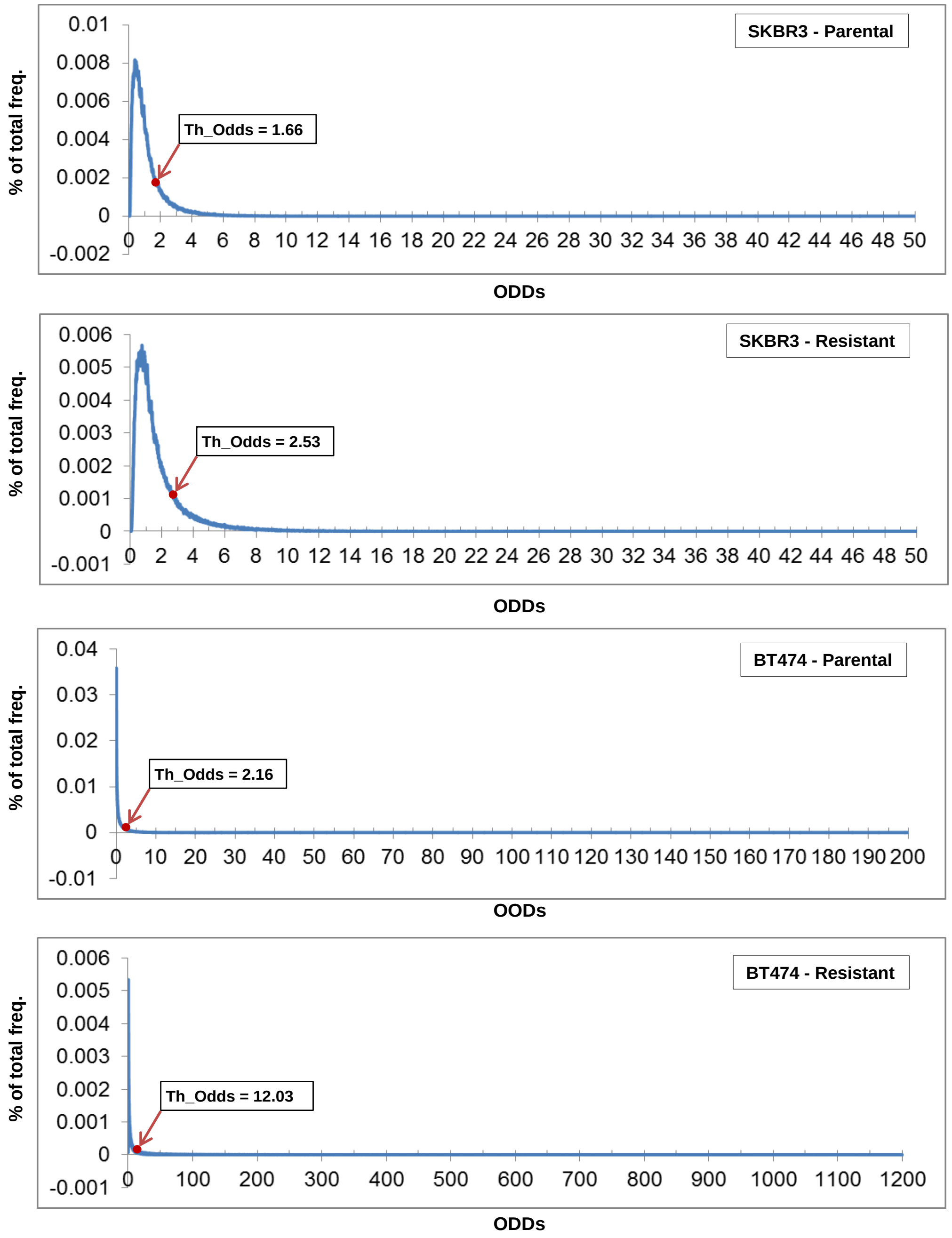

Figure 2. Frequency distributions of odds ratio values of gene-pairs in all four cases: SKBR3-Parental, SKBR3-Resistant, BT474-Parental, and BT474-Resistant after Bayesian analysis. In all four cases, selected thresholds of odds ratios, '*th_odds*' are shown with arrows which demarcated top 20% of pairs in their respective distributions.

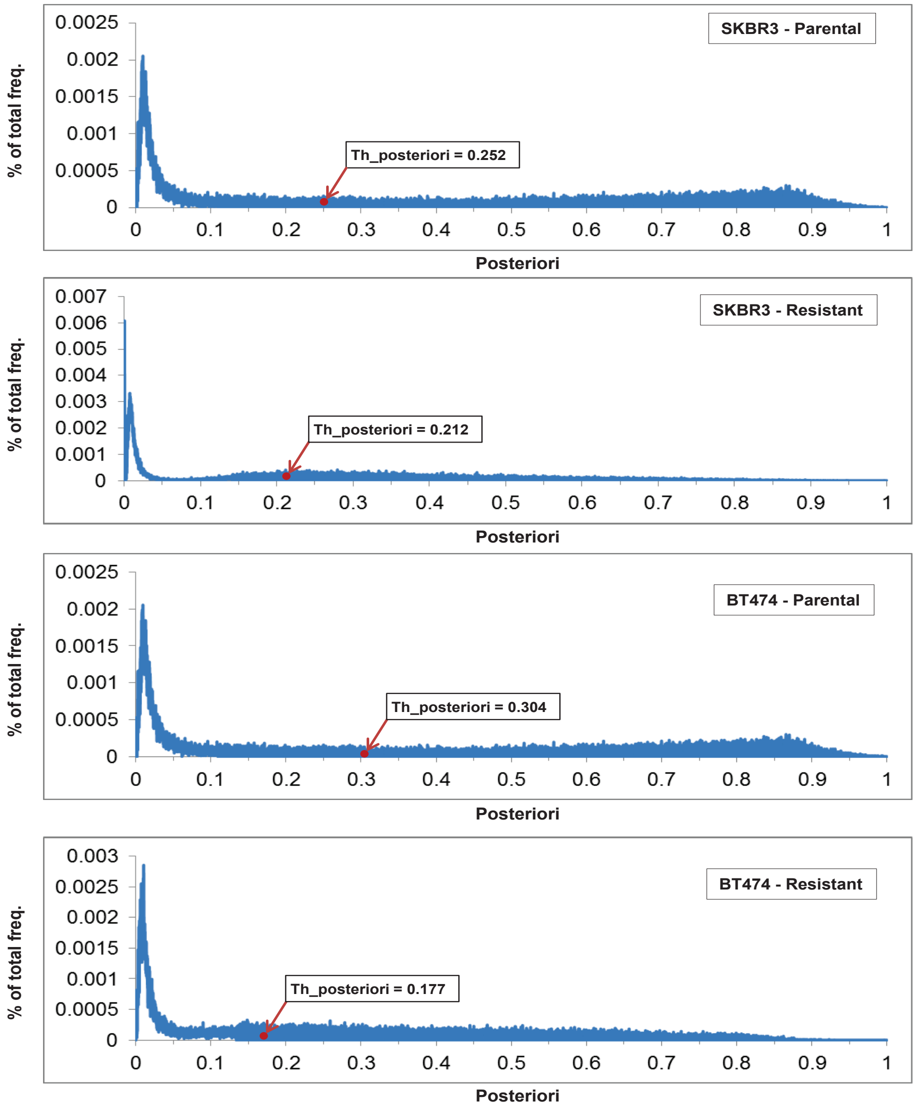

Figure 3. Frequency distributions of posteriori probabilities of gene-pairs that were selected after applying '*th_odds*' thresholds in all four cases: SKBR3-Parental, SKBR3-Resistant, BT474-Parental, and BT474-Resistant after Bayesian analysis. In all four cases, selected thresholds of posterior probabilities, '*th_posteriori*' are shown with arrows which demarcated top 50% of pairs in their respective distributions.

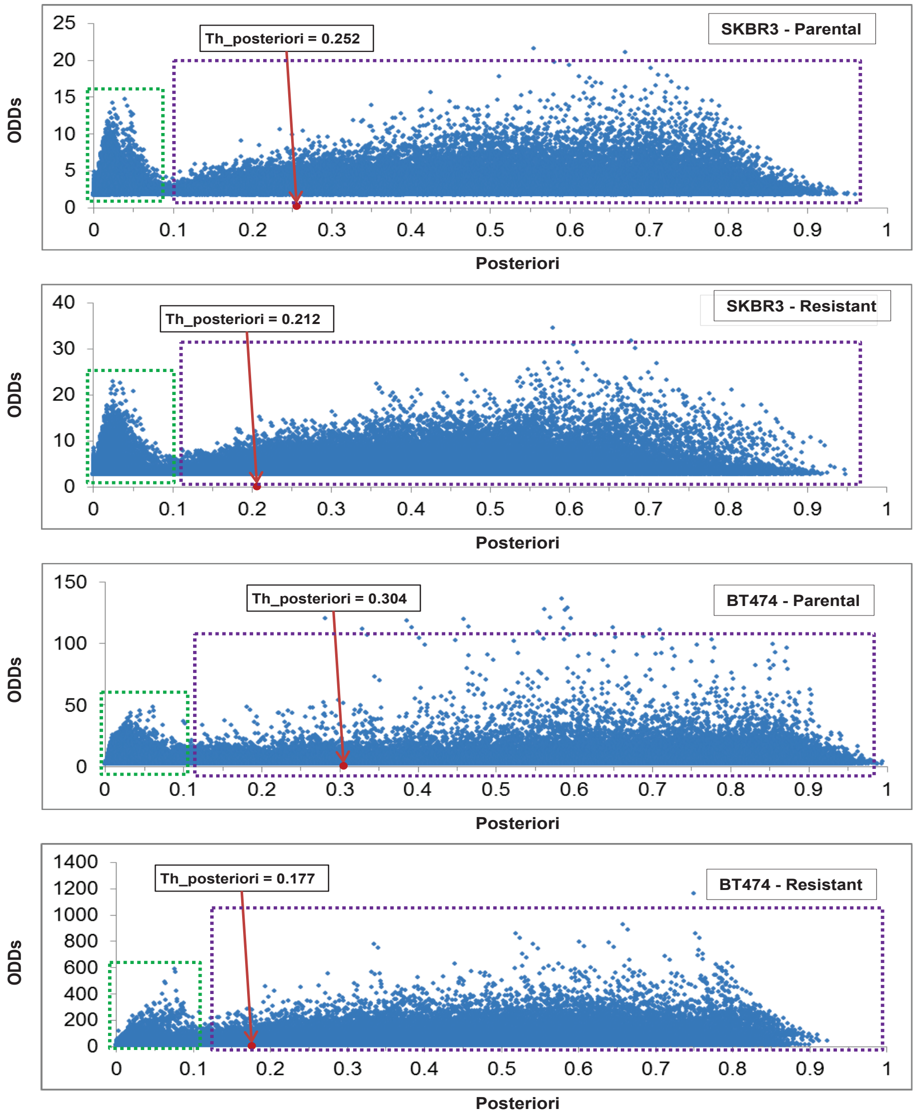

Figure 4. Scatter plots of *ODDs* VS *posterior probabilities* of gene-pairs that were selected after applying '*th_odds*' thresholds in all four cases: SKBR3-Parental, SKBR3-Resistant, BT474-Parental, and BT474-Resistant after Bayesian analysis. In all four cases: 1) two distinct distributions were shown within two boxes (in green and violet color), 2) selected thresholds of posterior probabilities, '*th_posteriori*' are shown with arrows which demarcated top 50% of pairs in their respective frequency distributions [See Supplementary Figure 3]
